# SMAD4 Expression in Renal Cell Carcinomas Correlates With a Stem-Cell Phenotype and Poor Clinical Outcomes

**DOI:** 10.3389/fonc.2021.581172

**Published:** 2021-05-03

**Authors:** Arezoo Rasti, Zahra Madjd, Leili Saeednejad Zanjani, Sadegh Babashah, Maryam Abolhasani, Mojgan Asgari, Mitra Mehrazma

**Affiliations:** ^1^ Oncopathology Research Center, Iran University of Medical Sciences (IUMS), Tehran, Iran; ^2^ Cellular and Molecular Research Center, Iran University of Medical Sciences (IUMS), Tehran, Iran; ^3^ Department of Basic Sciences/Medical Surgical Nursing, Faculty of Nursing and Midwifery, Tehran University of Medical Sciences (TUMS), Tehran, Iran; ^4^ Department of Molecular Medicine, Faculty of Advanced Technologies in Medicine, Iran University of Medical Sciences (IUMS), Tehran, Iran; ^5^ Department of Molecular Genetics, Faculty of Biological Sciences, Tarbiat Modares University (TMU), Tehran, Iran; ^6^ Hasheminejad Kidney Center, Iran University of Medical Sciences (IUMS), Tehran, Iran

**Keywords:** renal cancer, cancer stem cells, targeting, TGFβ signaling pathway, epithelial to mesenchymal transition

## Abstract

Renal cell carcinoma (RCC) is the most lethal neoplasm of common urologic cancers with poor prognoses. SMAD4 has a principal role in TGF-β (Transformis growth factorβ)-induced epithelial to mesenchymal transition (EMT) as a key factor in gaining cancer stem cell (CSC) features and tumor aggressiveness. This study aimed to evaluate the expression patterns and clinical significance of SMAD4 in RCC and the impact of its targeting on stem cell/mesenchymal cells and EMT characteristics in renal spheroid derived cells (SDCs) compared to parental cells (PCs) in RCC. The expression pattern and clinical significance of SMAD4 was evaluated in RCC. SDCs were enriched using a sphere culture system. Then SDCs and their PCs were compared with respect to their sphere and colony formation, expression of putative CSC markers, invasiveness as well as expression of genes, including stemness/mesenchymal, SMAD4 and TGFβ1genes. Finally, the effect of SMAD4 knockdown on SDCs was analyzed. We demonstrated that SMAD4 is positively correlated with decreased disease specific survival (DSS) in RCC patients and clear cell RCC (ccRCC) subtype and associates with poor DSS in patients with RCC, especially in ccRCC as the most metastatic RCC subtype. SDCs exhibited higher stem cell/mesenchymal properties. Inhibition of SMAD4 in PCs accelerated the dissociation of SDCs and decreased their clonogenicity, invasiveness, expression of mesenchymal markers and expression of SMAD4 and TGFβ1 genes compared to SDCs before transfection. We suggest that targeting SMAD4 may be useful against renal CSCs and may improve RCC prognosis.

## Introduction

Renal cell carcinoma (RCC) is the most popular epithelial malignancy of human adult kidneys and has the highest mortality among all urologic cancers (1). Over the last decade, the development of targeted molecular therapies has surprisingly improved the prognosis for patients with metastatic RCC, which is resistant to both radiotherapy and conventional chemotherapy and has a low clinical response rate (2). Despite advances in developing these targeted therapies, the 5-year survival rate for patients with metastatic RCC is only 12% and the overall survival (OS) in advanced metastatic RCC cases remains dismal (3).

Based on the cancer stem cell (CSC) model, a small subset of cancer cells have stem cell properties, such as expression of stem cells markers, clonogenic ability, lack of differentiative epithelial markers and sphere formation in the absence of adhesive cell culture medium (4). They are responsible for disease progression, tumor development, metastasis, recurrence and resistance to anticancer therapy (5). Recently, a population of CSCs with mesenchymal properties has been identified in renal human carcinomas (6). Furthermore, it has been proved that epithelial to mesenchymal transition (EMT) has a key role in getting CSC features in self-renewing SDCs from RCC cell lines (7). EMT is associated with the disruption of the intercellular junctions, gaining of a migratory mesenchymal-like characteristic, increased expression of stem cell-related transcription factors, tumorigenic ability, and tendency to be aggressive (2). EMT is getting started by the activation of transforming growth factorβ (TGFβ) pathway, which is the major deriving force for EMT (8). In late stage cancers, tumor cells often miss the ability to respond to TGF-β–mediated growth inhibition and instead apply TGF-β signaling to raise EMT invasion and metastasis (9).

SMAD4 is a member of Smad proteins, a family of intracellular mediators that regulate TGF-β superfamily signaling. It serves as a convergent node in the Smad pathways that are downstream of the TGF-β superfamily receptors (TGF-β RI and RII) and able to interact with all the R-Smad proteins to bind specific DNA sequence motifs and adjust the expression of target genes (10). SMAD4 has a key role in the tumorigenesis of many human cancers, including prostate, colorectal, and gastric cancers (11) and its dysregulation has also been found in few types of cancers (12). Nuclear SMAD protein complexes, including SMAD4, have all been mentioned as mediators of some or all phenotypic aspects of TGF- β-induced EMT in epithelial cells from kidney tubules, epidermis, and mammary glands (13). The activation of Smad2/3 and SMAD4 is a main regulatory mechanism in liver CSC self-renewal and stemness (14). Furthermore, the key role of SMAD4 in enhancing EMT activation and stem cell-like properties of spheres derived from RCC cell lines has been suggested by Lichner Z et al. (7). However, the molecular mechanism underlying the formation and survivance of self-renewing stem cells and the role of SMAD4-mediated TGF-β signaling in renal cancer progression and invasion is not completely clear.

Development of targeted molecular therapies has fundamentally improved the prognosis for patients with metastatic RCC over the last decade. These therapies include receptor tyrosine kinase inhibitors, monoclonal antibodies, and mammalian target of rapamycin (mTOR) inhibitors (15). These agents are mostly directed against angiogenesis-related signaling pathways (16). Many strategies are being developed to target the TGF-β pathway as a treatment for metastatic cancers (17).

To create more effective therapies, novel cellular pathways that may be involved in disease progression should be explored. To address this issue, we enriched cancer spheres from a metastatic RCC cell line with the use of sphere culture system and evaluated various biological characteristics. In addition, we examined the following hypothesis: Silencing SMAD4 as an important mediator of TGF-β pathway contributes to suppression of stem cell/mesenchymal cells and EMT characteristics in SDCs. Furthermore, for the first time, we compared the expression levels of SMAD4 between PCs and SDCs and examined the usefulness of nuclear SMAD4 expression as a prognostic marker in different RCC subtypes.

## Materials and Methods

### Patient Characteristics and Tumor Samples

RCC tissue samples were collected from Hasheminejad Kidney Center, a major university-based referral urology-nephrology center in Tehran, Iran, from 2007 to 2015. A total of 209 patients who had undergone radical nephrectomy and had no history of preoperative radiation or hormone therapy were included in this study. The samples comprised various subtypes of RCC, including clear cell, chromophobe, and papillary RCC.

Medical archival records and hematoxylin eosin (H&E) stained slides were retrieved to obtain clinicopathological parameters, including age, gender, tumor size (maximum tumor diameter), tumor stage, and nucleolar grade.

In addition, the presence of distant metastasis, necrosis, regional lymph nodes involvement, renal vein, pelvis and sinus, Gerota’s fascia, and microvascular invasion (MVI) were recorded. Disease-specific survival (DSS) was defined as the time from radical nephrectomy to the date of death related to the patient’s cancer.

### Immunohistochemistry (IHC) and Immunostaining Evaluation

Construction of tissue microarrays (TMAs), immunohisto-chemistry (IHC) staining, immunostaining evaluation, and scoring system were done (18). TMA blocks were constructed in 3 copies, each containing 1 sample from a different regions of the tumor; tissue sections were then incubated overnight at 4°C with the anti-SMAD4 antibody (sc-29484, Santa Cruz Biotechnology) using a 1:100 dilution. After being washed 3 times in Tris-buffered saline (TBS), sections were incubated with Mouse/Rabbit UnoVueTM HRP/DAB detection system, (UMR100PD, UMR1000PD, Diagnostic BioSystems, Netherlands) as the secondary antibody for 15 minutes. For negative controls, the primary antibody step was replaced with TBS and only the secondary antibody was used. Human breast cancer tissues were used as a positive control for SMAD4 staining. The cutoff point of 200 was selected based on the median H-score to categorize the samples as high or low nuclear SMAD4 expression.

### Cell Line

ACHN cell line was purchased from Iranian Biological Resource Center (IBRC). Cells were preserved in standard medium, consisting of Dulbecco’s modified Eagle’s medium (DMEM [supplemented with 10% heat-inactivated (56°C, 30 min) fetal bovine serum (FBS) with 2 mM L-glutamine, 2 mM nonessential amino acids (NEAA), 100 U/mL penicillin, and 100 µg/mL streptomycin in a humidified atmosphere at 37°C under 5% (CO2)]. All from Gibco, Invitrogen, USA).

### Sphere Formation Assay

To evaluate sphere-formation potential, single viable cells at a density of 7 × 10^5^ from PCs were plated in serum-free defined media (SFDM) at pre transfection and post transfection with SiSMAD4. They consisted of the mentioned mediums supplemented with L-glutamine, penicillin/streptomycin, 20 ng/ml basic fibroblast growth factor (bFGF; Peprotech, USA), and 10 ng/ml epidermal growth factor (EGF; Peprotech, USA); also, poly hydroxyethyl methacrylate (poly-HEMA) (Sigma, USA) coated flasks were used to inhibit cell adhesion (19). Fresh aliquots of bFGF and EGF were added every other day for cultures up to 10 to 12 days. SDCs were gathered by gravity, washed with PBS, and detached with trypsin (Gibco, Invitrogen, USA); then, they were transferred into the cell culture coated flask to promote further generations. Next, single viable cells (1000 cells/dish) from PCs and SDCs collected from the first generation of spheroids were seeded in serum-free medium including 10 ng/ml EGF and 20 ng/ml bFGF, which were added every other day into plates with the use of ultra-low-attachment 6-well plates (Corning, Costar, USA). After 12 days, the spheroid number of each well was counted and photographed with an Olympus fluorescent microscope.

### Colony Formation Assay

A total of 120 single viable cells from SDCs and their PCs were seeded into 6-well culture plates (Corning, Costar, USA), containing 2 mL DMEM supplemented with 10% FBS, and were allowed to grow for 10 days at 37°C. The cell colonies were fixed with 4% paraformaldehyde (Merck, Germany) and stained with 0.05% crystal violet (Sigma, USA). The number of colonies with more than 50 cells adhering to the bottoms of the plates was counted blindly under the microscope (×200) in all fields.

### Cell Invasion Assay

By following the manufacturer’s recommendations, we performed *in vitro* trans well invasion assay by Cultrex BME Cell Invasion Assay kit (R&D Systems, USA).

SDCs and their PCs were suspended in serum-free DMEM at a concentration of 5×10^4^ cells/mL for 18-24 hours before the assay. Then, the medium was gathered and the cells were re suspended at 1 x 10^6^ cells/mL in serum-free medium. The upper chamber was loaded with 50 μL cell suspension and the lower chamber with 150 μL DMEM with 5% FBS. The remaining cells were applied for the standard curve based on the manufacturer’s protocol. The top and bottom chambers were aspirated and washed following a 48-hour culture. Then, Calcein AM solution mixed with cell dissociation solution was added to the bottom chamber and incubated at 37°C for 1 hour. The plate was read at 485 nm excitation and 520 nm emission (Bio Tek, USA). The standard curve was used to determine the number of invaded cells the percentage of cell invasion as well as (**Supplementary Appendix, S1**).

### Flow Cytometric Analysis

Flow cytometry was used to compare the protein expression of a panel of suggested renal CSCs and differentially expressed markers between SDCs and their PCs in other cancers (20). The following fluorophore-conjugated mouse antihuman monoclonal antibodies were used for flow cytometry: phycoerythrin (PE)-labeled mouse antihuman CD24, fluorescein isothiocyanate (FITC)-labeled mouse antihuman CD44, PE-labeled mouse antihuman CD133, PE-labeled mouse antihuman CD105, PE-labeled mouse antihuman CXCR4, PE-labeled mouse antihuman CD56, FITC-labeled mouse antihuman CD90, PE-labeled mouse antihuman CD29, PE-labeled mouse antihuman CD73, FITC-labeled mouse antihuman CD34, PE-labeled mouse antihuman CD146 and PE-labeled mouse antihuman CD117 (All antibodies were from BD Biosciences, USA, except CD90 from DAKO, Denmark).

Also, 1×10^5^ single viable cells from live cells of PCs and SDCs were dissociated with accutase (Sigma-Aldrich, St. Louis, MO, USA) washed once with PBS, and stained with conjugated antibodies, or respective isotype controls according to the manufacturers’ recommendations. Then, the cells were washed, re suspended, and analyzed with FACSCalibur (Becton Dickinson, San Jose, CA). The FlowJo Version 7 was also used for data analysis.

### RNA Isolation and Quantitative Real Time PCR (qRT-PCR)

Total RNA was extracted using RNeasy kit (Qiagen), treated with DNase I, and reverse transcribed with Transcriptor First Strand cDNA Synthesis Kits (Bioneer and PARS GENOME). Also, qRT-PCR was performed by means of the Selected SYBR Green master mix (TAKARA) on an ABI Step One Detection System *via* the following program: 95°C for 3 minutes, 39 cycles alternating in turn with 95°C for 15 seconds, 60°C for 1 second, and 72°C for 1 minute, and maintained at 75°C for 5 minutes. PCR primers are listed in **Supplementary Table S2** (**Table S2**). Gene expression was quantified based on the CT value and normalized to the levels of U6 small nuclear RNA (U6 snRNA) for miR-204 gene and GAPDH for another assessment.

### Western Blotting

PCs and SDCs were lysed on ice in RIPA buffer (25mM Tris-HCL pH 7.6, 150mM NaCI, 1% NP40, 1% sodium deoxycholate, 0.1% SDS, 1mM benzamidine, 2ug·mL-1 pepstatin A, 2ug·mL-1 leupeptin, 2ug·mL-1 aprotinin and 0.5mM PMSF for 30 minutes. Samples were centrifuged at 2000 g for 15 minutes and protein content was measured by Biuret assay. Equal amounts (10 ug) of total proteins were denatured in 4× sodium dodecyl sulfate (SDS) loading buffer (1 mM Tris·HCl, pH 6.8, 1% SDS,1% 2-mercaptoethanol, 10% glycerol), boiled for 5 minutes, separated by electrophoresis on 12% SDS-polyacrylamide gels, and transferred to poly vinylidene difluoride (PVDF) membranes. After blocking with 5% nonfat milk and 0.05% Tween 20 in TBS for 1 hour, the membranes were incubated at first with mouse monoclonal anti-SMAD4 (sc-29484, Santa Cruz Biotechnology) as a specific primary antibody at a dilution of 1:1000. Then, horseradish peroxidase-conjugated secondary antibody (sc-516102, Santa Cruz Biotechnology) (1:5000 dilution) was visualized by ECL detection system. Also, β-actin was applied as a loading control.

### RNAi Knockdown of SMAD4 Expression

Synthetic double stranded siRNAs designed to target human SMAD4 (siSMAD) and silencing negative control (siCtrl) were purchased from Santa Cruz Biotechnology. PCs were grown on 6-well plates in normal growth medium without antibiotics and transfected with siSmo and siCtrl at the final concentration of 50 nmol/L using Lipofectamine RNAiMAX (Invitrogen) according to the manufacturer’s protocol. The efficacy of transfection was analyzed 24 hours post transfection and was confirmed by flow cytometry. After 24, 48, and 72 hours, transfected cells were harvested to determine the siRNA knockdown efficiency by quantitation of SMAD4 expression *via* q-RT-PCR. After 48 hours, PCs with the lowest SMAD4 gene expression level were used for sphere formation assay. The single cells obtained from these spheres were applied for the following techniques: sphere formation, flow cytometry, genes expressions, Western blot, and invasion assay.

### Statistical Analysis

All experiments were carried out in triplicate. The data were expressed as mean ± standard deviation (SD). Statistical analysis was done using unpaired 2-tailed Mann–Whitney U test or the student’s t test in the “IBM Corp. Crosstab analysis was used to determine the association of SMAD4 with clinicopathological parameters. Also, Kaplan–Meier method was used to determine DSS curves, and log-rank test was utilized to compare the estimated curves between groups. Univariate and multivariate analysis were done using the Cox proportional hazards model. SMAD4 levels larger than the median values were classified as high.

## Results

### SMAD4 Nuclear Expression Is Associated With Worse Prognosis and May Serve as a Novel Prognostic Marker in ccRCC Patients

In this study, using an immunohistochemical technique, we tested the expression of SMAD4 in RCC samples to investigate the clinicopathologic association of SMAD4 expression and to assess the prognostic value of SMAD4 in RCC.

### Patient Characteristics

Of the 209 RCC samples, 158 (75.6%) were ccRCC, 34 (16.3%) pRCC, and 17 (8.1%) chRCC. The main demographic characteristics of patients are provided in **Supplementary Table 3** (**Table S3**).

### Comparison of SMAD4 Expression in RCC Subtypes

**Figure 1:**
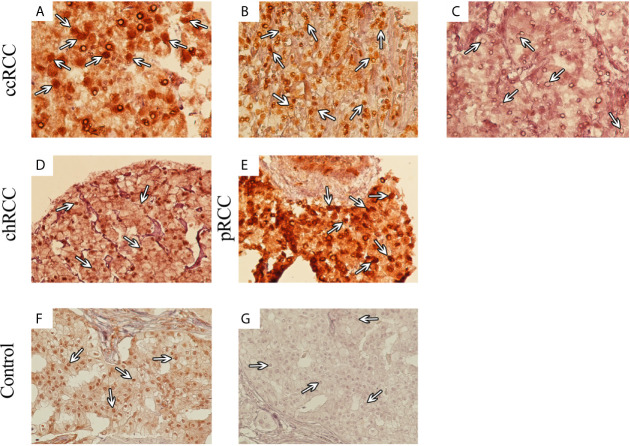
Immunohistochemical (IHC) Analysis of Nuclear SMAD4 Expression in Different 707 Renal Cell Carcinoma (RCC) Samples. RCC samples expressed SMAD4 at various levels. SMAD4 expression in clear cell RCC at various levels: weak **(A)**, moderate **(B)**, and strong **(C)**. Moderate expression of SMAD4 in chromophobe RCC **(D)** and strong expression of SMAD4 in papillary RCC **(E)**. IHC staining of breast cancer tissue as positive **(F)** and negative **(G)** controls. **(A–C)** are presented with a magnification of 400×, **(D–G)** are presented with a magnification of 200×).

Analysis of TMA-based IHC staining demonstrated that expression of SMAD4 was localized to the nucleus and cytoplasm of tumor cells. Based on the translocation of SMADs from cytoplasm to the nucleus, their action as transcriptional factors in the nucleus (21) and considering our observation about cytoplasmic expression of SMAD4 in normal renal tissues, the nuclear expression of SMAD4 was considered for evaluation. In RCC, the nuclear expression of SMAD4 was 95.2% (199/209) with varying intensity levels. IHC analysis of nuclear SMAD4 expression in different RCC samples is presented in **Figure 1**.

### Association of SMAD4 Expression With Clinicopathological Parameters in RCC

The association of SMAD4 expression with clinicopathological characteristics in RCC is summarized in **Table S3**. Also, a statistically significant difference between the nuclear expression of SMAD4 in different RCC subtypes was observed (*P* value 0.034). Expression of SMAD4 was significantly associated with age and renal pelvis involvement (*P* values 0.046 and 0.04, respectively).

### Prognostic Significance of SMAD4 Expressions

**Figure 2:**
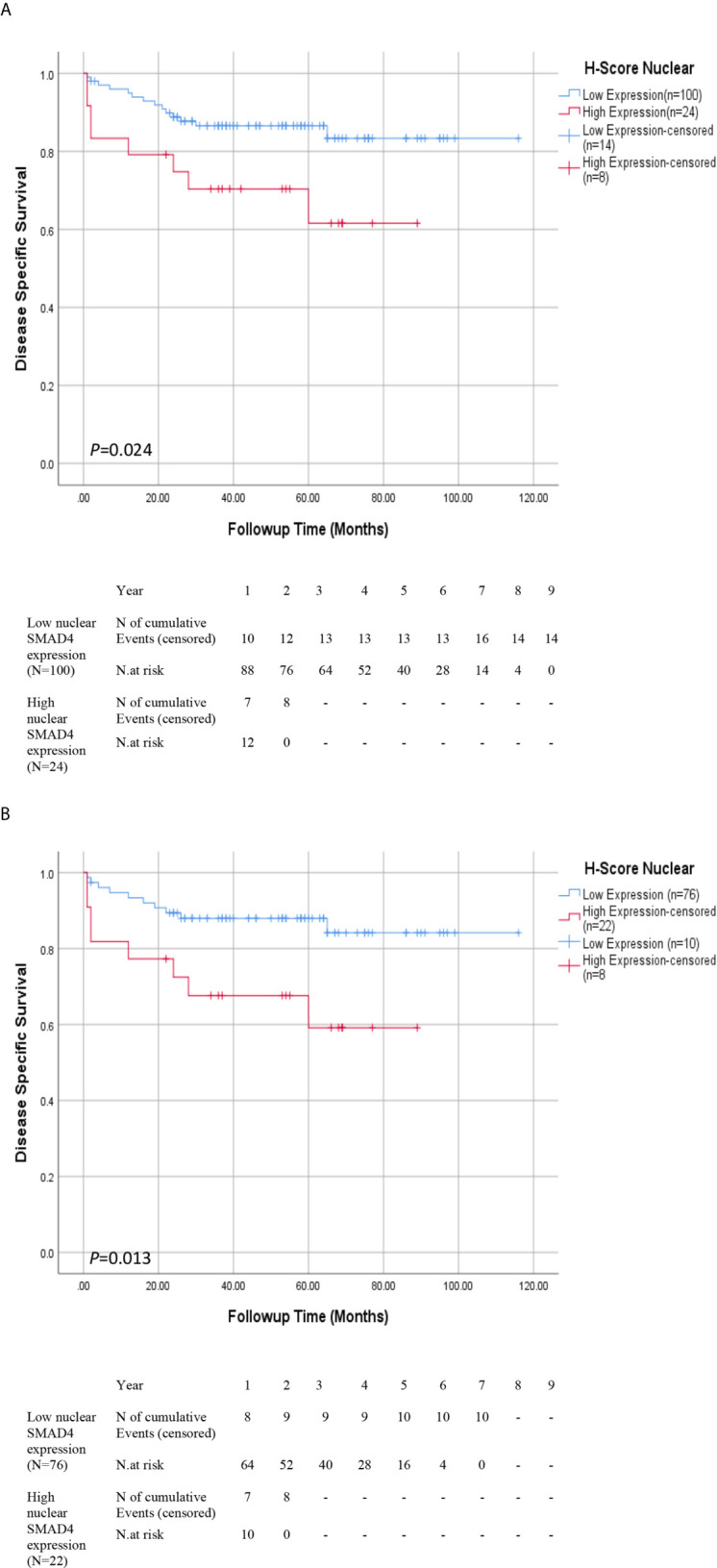
Association Between Expression of SMAD4 and Survival Rates in Patients With Renal Cell Carcinoma (RCC). Disease-specific survival (DSS) with nuclear SMAD4 expression grouped into high versus low expression levels in **(A)** RCC and **(B)** ccRCC patients. Number of events and at risk patients on the Kaplan Meier estimates are presented underneath the graphs **(A, B)**.

The median follow-up time of surviving patients was 47.0 months, ranging from 1–116 months. During the follow-up period, disease-related death occurred in 22 patients (10.5%). The 5-year DSS survival rates was 83.0% in low nuclear SMAD4 expression and 57% in high nuclear SMAD4 expression. Nuclear overexpression of SMAD4 was associated with shorter DSS survival than low expression group (*P* = 0.024) (**Figure 2A**).

The results of the Cox proportional univariate and multivariable analysis of the relationships between prognostic variables and survival are presented in **Table 1**. The results of the multivariable analysis showed that nucleolar grade, nuclear SMAD4 expression, tumor stage, and tumor size were significant risk factors affecting the DSS of patients with RCC. Nuclear SMAD4 expression and tumor size showed independent poor DSS, with hazard ratios of 3.10 and 1.13 and *P* values 0.013 and 0.034, respectively.

**Table 1:** Univariate and multivariable analysis of disease free survival (DSS) and progression-free survival (PFS) in patients with renal cell carcinoma (RCC).

Feature	DSS			
	Univariate		Multivariable	
	HR (95%CI)	*P* value	HR (95%CI)	*P* value
**Age**				
(>55.0 vs.≤55.0)	1.16 (0.50-2.69)	0.723	–	–
**Sex**				
(male vs. female)	0.92 (0.36-2.37)	0.877	–	**-**
**Clinical stage**				
(III/IV vs. I/II)	1.79 (1.11-2.87)	**0.016**	1.10 (074-2.29)	0863
**Nucleolar grade**				
(III/IV vs. I/II)	3.38 (1.39-8.25)	**0.007**	2.57 (0.96-6.83)	0.058
	
**Tumor size**	1.16 (1.04-1.28)	**0.005**	1.13 (1.01-1.26)	**0.034**
**Nuclear SMAD4 Expression**				
High vs. low	2.60 (1.09-6.21)	**0.031**	3.10 (1.26-7.61)	**0.013**

HR, hazard ratio; CI, confidence interval; MVI, microvascular invasion.

Values in bold are statistically significant.

### Association of Nuclear Expression of SMAD4 With Survival Outcomes in ccRCC

DSS and Cox proportional univariate and multivariable analyses were carried out only for ccRCC patients due to limited number of occurred events in ChRCC and pRCC subtypes. The results of Kaplan-Meier survival analysis revealed that ccRCC patients whose tumors expressed higher nuclear levels of nuclear SMAD4 showed significantly poorer DSS than those other phenotypes expressions (*P* = 0.013) (**Figure 2B** and **Supplementary Table S4**).

Nuclear SMAD4 expression and tumor size were significant independent risk factors affecting the DSS of patients with ccRCC in multivariable Cox proportional analysis, with hazard ratios of 3.70 and 1.71 and *P* values 0.006 and 0.008, respectively.

### SDCs Formed Stable Spheres, With a Higher Clonogenic Potential, Larger Colony Size, and Increased Invasion Potential Compared to PCs

**Figure 3:**
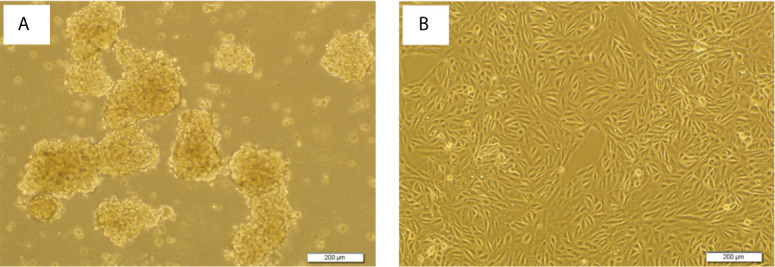
Sphere formation by ACHN cells. Sphere Formation by ACHN Cells. **(A)** Spheroid derived cells (SDCs) from parental ACHN cells (PCs) cultured anchorage-independent conditions formed typical spheroids in the presence of growth factors at the second passage. **(B)** ACHN adherent monolayer cells. Figures are shown with a magnification of 100 X.

**Figure 4:**
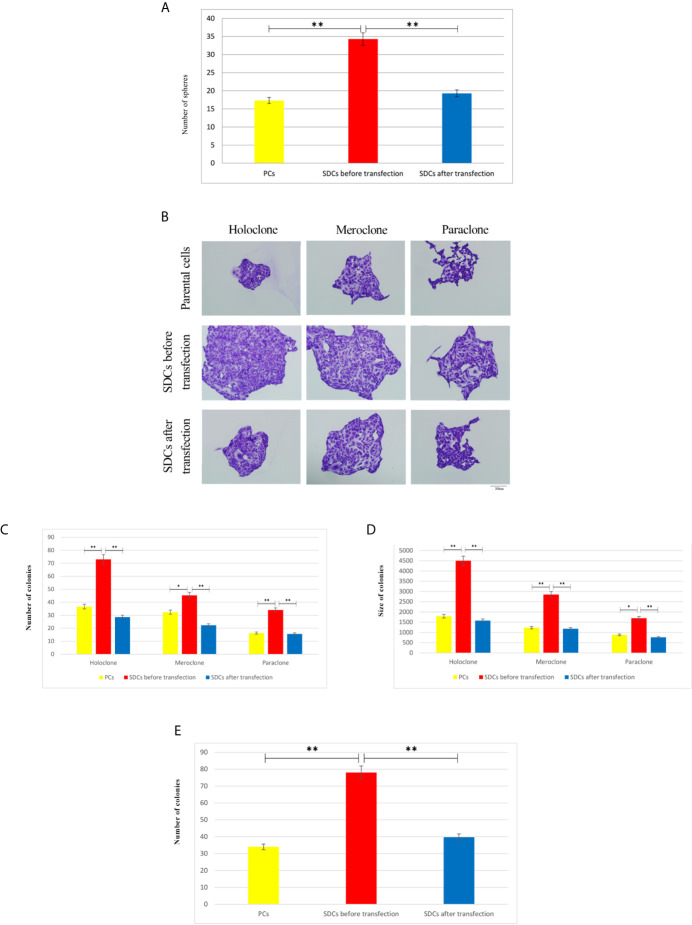
Clonogenicity and Sphere Formation Potential of Parental Cells (PCs) and Spheroid-Derived Cells (SDCs). **(A)** Sphere formation capacity of SDCs was significantly higher compared to PCs and was decreased in SDCs after transfection with SiSMAD4 (*P* < 0.01). The colony and sphere numbers were counted under a dissection microscope. **(B)** Three types of colonies termed holo, mero, and paraclones were identified during the colony formation assay in 3 cell populations, including PCs and SDCs before and after transfection with SiSMAD4. **(C, D)** The potential of clonogenicity was significantly higher in SDCs than PCs and was significantly decreased in SDCs after transfection with SiSMAD4. **(E)** The final results of cell invasion assay showed that SDCs were significantly more invasive (2.27-fold) than PCs, and invasive potential was significantly decreased (1.96-fold) in SDCs after transfection of PCs with SiSMAD4. Data are represented as mean ± SD (n = 3 each). **P < 0.01. (n = 3 each) and *P < 0.05.

The ability of cell lines to form SDCs *in vitro* is related to the presence of a self-renewing cell population (22). Initially, the ability to form spheres from metastatic renal cancer cell line (ACHN) was tested. SDCs formed free-floating cellular aggregates and compact no adherent spheres in serum-free medium in the presence of 10 ng/mL EGF and 20 ng/mL bFGF after 10 to 12 days (**Figure 3**). SDCs were also capable of being sub cultured; therefore, the second generation of these SDCs were used for other experiments. In addition, PCs, a significantly lower sphere forming ability than SDCs (*P* < 0.01) (**Figure 4A**).

### SDCs Presented Increased Clonogenic Potential, With Larger Colony Size, and Increased Invasion Potential Compared to PCs

To evaluate the CSC properties of SDCs, their clonogenicity, regeneration ability, and colony size were compared to PCs. Cancer cells displayed all 3 types of colony, (holoclone, meroclone, and paraclone) with the holoclone fraction being enriched in CSCs. Meroclones and paraclones had lower growth potential than holoclones (**Figure 4B**). Our findings showed that there were statistically significant differences in colony-forming ability between SDCs and their PCs with respect to the mean colony size and number of colonies (**Figures 4B–D** and **Supplementary Table S4**).

We carried out a cell invasion assay to explore the invasive properties of PCs and SDCs. Results showed a lower invasive potential in PCs compared to SDCs (*P <*0.01) (**Figure 4E** and (**Supplementary Appendix, S1**).

### SDCs Formed Stable Spheres With Stem Cell/Mesenchymal Properties

**Figure 5:**
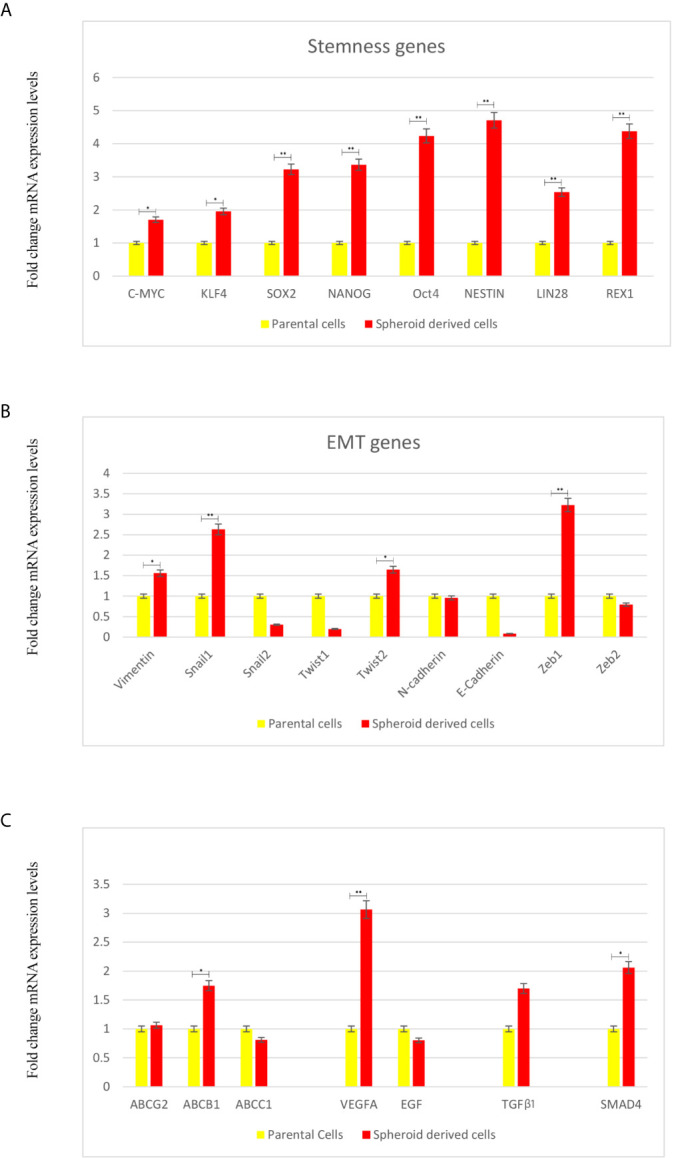
Expression Levels of Stemness, EMT, and Angiogenesis-Related Genes and TGF-β1 using qRT-PCR in Parental Cells (PCs) and Spheroid-Derived Cells (SDCs) From Before and After Transfection of PCs with SiSMAD4. **(A)** Stem cell marker, **(B)** Mesenchymal marker, **(C)** ABC transporters, Angiogenesis-related and TGF-β1 genes expression of PCs and SDCs in SFDM and SDCs established by SMAD4 inhibition were quantified by qRT-PCR. Data are represented as mean ± SD (n = 3 each). *P < 0.05, and **P < 0.01.

Next, we evaluated if these SDCs have other stem cell-related characteristics. Our results indicated a significant 1.7-4.7-fold increase of all stem cell markers in SDCs (*P <*0.05) (**Figure 5A**).

Whether a transition to mesenchymal phenotype was associated with increased stem cell marker levels was also tested. We observed a significant increase in Zeb1 and Snail 1 (*P <*0.01), Twist2 and Vimentin (*P <*0.05). On the other hand, expression of the epithelial marker E-cadherin seemed to be upregulated in PCs relative to SDCs (**Figure 5B**).

In this study, we investigated ABC transporters genes (ABCB1, ABCC1 and ABCG2), VEGFA and EGF as angiogenesis related genes in SDCs and PCs. We observed a significant decrease in ABCB1 and VEGFA levels in PCs compared to SDCs (*P <*0.05). Considering the promoted role of TGFβ-1 in metastatic cancers (23), we investigated TGFβ-1 gene expression level. We observed a significant increase in TGFβ-1 in SDCs compered to PCs (*P <*0.05) (**Figure 5C**).

### SDCs Presented Higher Expression of Putative Cancer Stem Cell Markers Compared to Parental Cells

**Figure 6:**
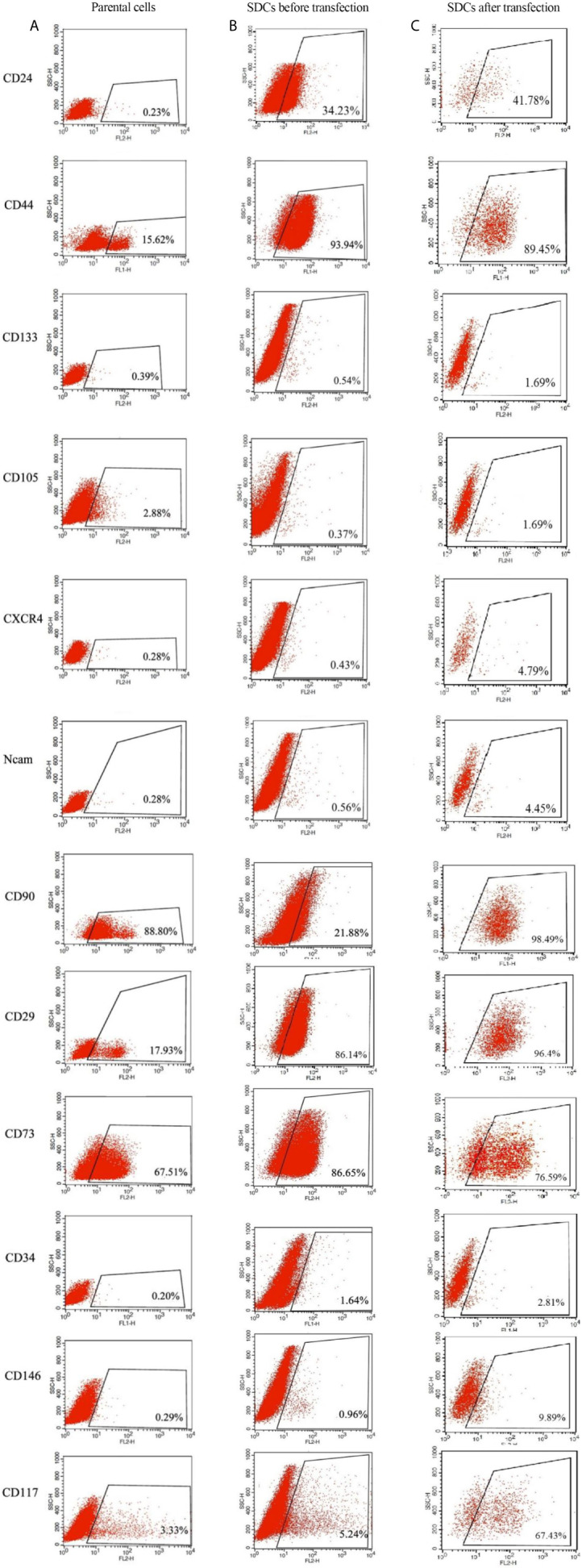
Expression Levels of Putative CSC Markers in Parental Cells (PCs) and Pre transfected and Post Transfected Spheroid-Derived Cells (SDCs) Using Flow Cytometry. **(A)** Expression of CSC markers in PCs. **(B)** Expression of CSC markers in SDCs before transfection with SiSMAD4. **(C)** Expression of CSC markers in SDCs after transfection with SiSMAD4. Data are represented as mean ± SD (n = 3 each).

To characterize and evaluate PCs and SDCs, the expression of putative CSC markers in the 2 cell populations was performed using the flow cytometry technique (**Figures 6A, B**). The alterations in surface markers in these spheres exhibit the tendency to CSC and mesenchymal phenotype compared to PCs.

### SMAD4 Expression Is Needed to Maintain SDCs and Its Knockdown Reduces the Formation of Cancer Spheres and Invasive Characteristics of Cancer Cells

**Figure 7:**
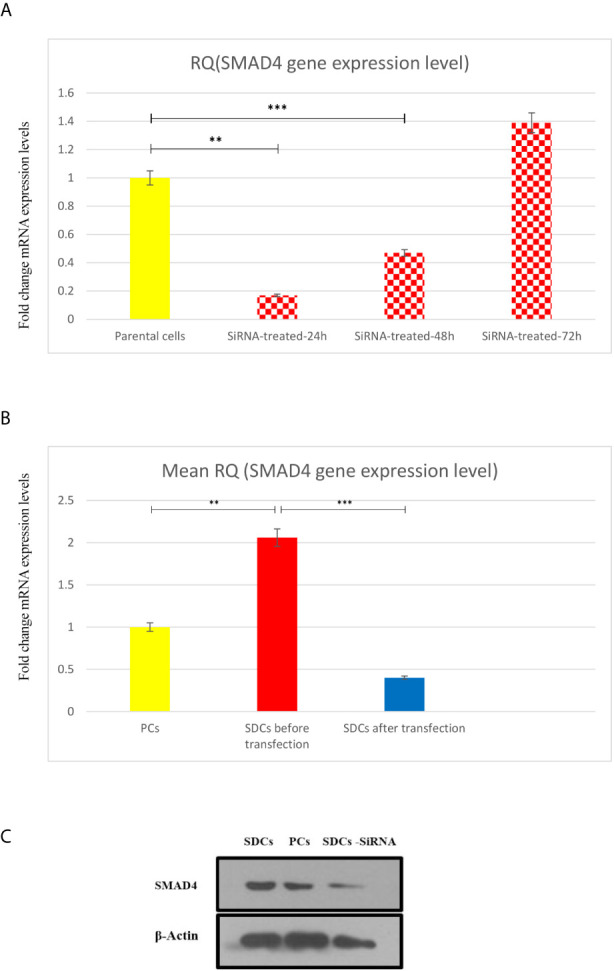
Transfection of Parental Cells (PCs) With SiSMAD4 and its Effect on Expression Level of SMAD4. **(A)** Relative SMAD4 mRNA level in PCs was measured 24,48 and 72 hours after SMAD4 knockdown compared to PCS. **(B)** SMAD4 gene expression level of PCs and SDCs before and after transfection with SiSMAD4. **(C)** Measurement of protein SMAD4 expression by Western blotting in PCs and SDCs before and after SMAD4 knockdown. Data are represented as mean ± SD (n = 3 each). **P < 0.05, and ***P < 0.0001.

Since SMAD4 has been suggested to have a key role in TGFβ signaling pathway in RCC spheres, and thus may contribute to EMT process (7), we analyzed the effect of SMAD4 knockdown on SDCs. The transfection efficiency was investigated by flow cytometry and fluorescence microscopy and the percentage of fluorescein‐labeled cells was calculated to be about 69% in both methods. Transfected cells were harvested to determine the siRNA knockdown efficiency after 24, 48, and 72 hours by quantitation of SMAD4 expression *via* qRT-PCR (**Figure 7A**). After 48 hours, PCs with the lowest SMAD4 gene expression level (0.27-fold change gene expression level compared to PCs) were used for sphere and colony formations, invasion assay, flow cytometry, genes expressions, and Western blot techniques.

The SMAD4 gene expression level was quantified in 3 populations (PCs and SDCs pre transfection and post transfection upon SMAD4 inhibition or treatment with transfection agent). As expected, SMAD4 mRNA levels were more than 2-fold higher in SDCs than in the corresponding PCs and had a highly significant decrease in post transfection SDCs (*P <*0.0001) (**Figure 7B**).

**Figure 8:**
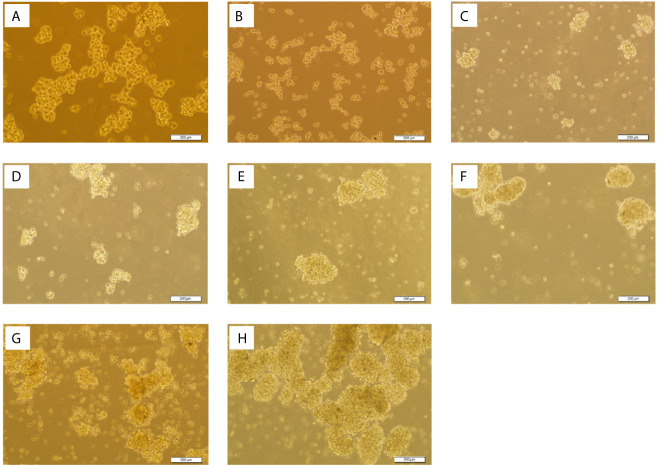
Spheroid Derived Cells (SDCs) With Stem Cell-Related Features Before and After SMAD4 Transfection. Sphere formation assay in serum-free defined medium (SFDM) compared to the self-renewal capacity 3D sphere derivatives of the PCs after SiSMAD4 inhibition during 6 days (**A**:1st to **G**:7th day). **(H)** SDCs before transfection with SiSMAD4 showed significantly higher sphere-forming ability after 10 days. Figures are shown with a magnification of 100×.

After confirming successful SiSMAD4 knock-down, we explored SMAD4 gene and protein expression by Western blot analysis in PCs and 2 SDCs populations. Interestingly, SMAD4 expression was higher in the SDCs before transfection, compared to the respective PCs, and it was significantly decreased in SDCs after transfection (**Figure 7C**). Furthermore, reduction in SMAD4 expression through siRNA treatment of PCs led to a decrease in sphere formation. Six days after culturing, post transfected SDCs lost their integration and became dissociated (**Figure 8**). In addition, SDCs generated a significantly lower sphere forming ability after transfection compared to before transfection (*P* < 0.01) (**Figure 4A**).

To establish the ability of SMAD4 inhibition to suppress self-renewal, we quantified the clonogenic ability of single cells of SDCs population pre transfection and post transfection upon SMAD4 knockdown with transfection agent.

Single cells of SDCs population formed smaller colonies and had lower colonization ability upon SMAD4 knockdown after transfection compared to the match cells before transfection (*P* < 0.05) (**Figures 4B–D** and **Supplementary Table S4**).

The cell invasion assay confirmed that invading cell numbers significantly decreased after SiSMAD4 transfection compared to the SDCs population (*P <*0.01) (**Figure 4C** and **Supplementary File, S1**).

### SMAD4 Knockdown Alters the Expression of Surface Markers, Reduces the Stem Cell/Mesenchymal Properties of Cancer Spheres, and Upregulates the miR-204 Expression in SDCs

Flow cytometric analysis demonstrated that after transfection upon SMAD4 knockdown, single cells of SDCs population had a significant increase in the expression of the epithelial marker of CD24 (41.78%) and CD133 (1.69%) and presented decreased expression of CD44 (89.45%) and CD73 (76.59), as mesenchymal markers with the match cells before transfection (**Figure 6C**).

**Figure 9:**
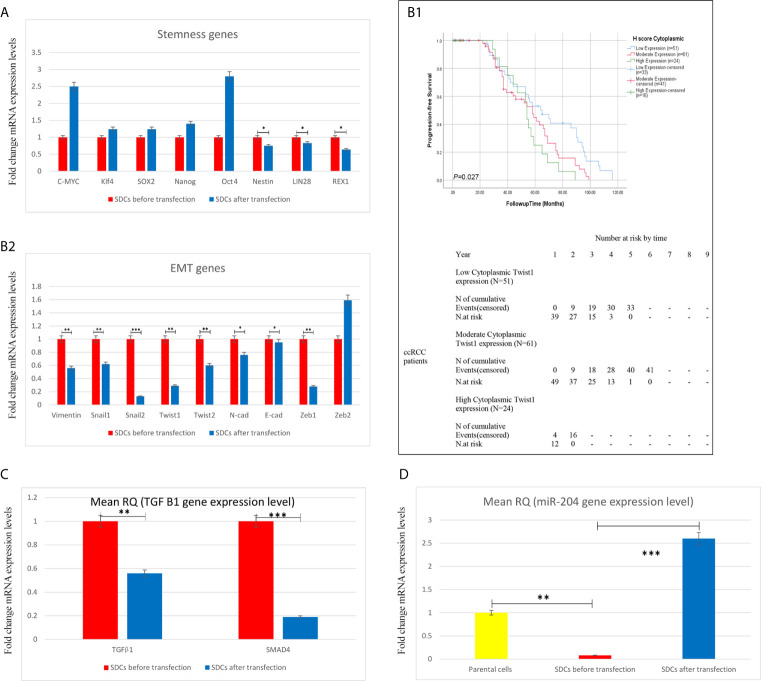
Expression Levels of Stemness, EMT, and Angiogenesis-Related Genes, TGF-β1 and miR204 using qRT-PCR in Spheroid-Derived Cells (SDCs) From Before and After Transfection of PCs with SiSMAD4, cytoplasmic Twist1 expression and survival rates in patients with clear cell Renal Cell Carcinoma. **(A)** Stem cell markers, **(B2)** Mesenchymal markers, **(C)** TGF-β1 gene and **(D)** (miR204) expression of SDCs in SFDM and SDCs before and after Transfection by SMAD4 inhibition were quantified by qRT-PCR. Data are represented as mean ± SD (n = 3 each). *P < 0.05, **P < 0.01 and ***P < 0.0001. **(B1)** Correlation between cytoplasmic Twist1 expression and survival rates in patients with clear cell Renal Cell Carcinoma (ccRCC). Progression-free survival (PFS) with cytoplasmic Twist1 expression grouped into low- versus moderate- versus high expression levels.

We performed qRT-PCR analysis to compare gene expressions in 2 SDCs populations. After transfection upon SMAD4 knockdown, single cells of SDCs population expressed significantly lower levels of stemness REX1, Nestin and Lin28, Twist1 (*P <*0.05) and other mesenchymal genes except Zeb2 and TGFβ1(*P <*0.01) compared to the match cells before transfection (**Figures 9A–C**). Because of the role of Twist1 in macrophage recruitment to tumor tissues and the role of tumor-associated macrophages (TAMs) in cancer progression and metastasis by stimulating tumor growth, angiogenesis, and cellular invasion, migration and EMT induction, we selected Twist1 and examined its cytoplasmic and nuclear expression in the same set of RCC tissues, by immunohistochemistry on a tissue microarray. Our data exhibited that increased cytoplasmic expression of Twist1 as an EMT-related transcription factor was associated with worse prognosis in more metastatic RCC subtypes and especially in ccRCC (24) (**Figures 9B 1&2** and **Supplementary File S5**).

In the study of Lichner, Z et al, the TGFBR2-SMAD2/3-mediated branch of the TGFβ pathway was predicted by miR Path and Target Scan programs to be targeted by several miRNAs (miR-200c, miR-204, miR-17, miR-218, miR-590-5p, miR-204, miR-18a, miR-18b, miR-186, miR-330 andmiR-886-3p) downregulated in RCC SDCs, and miR-204 was one of the most down regulated miRNAs which directly targets SMAD4 (7). On the other hand, the results of miRNA profiling in human PCO (posterior capsule opacification) tissues demonstrated that, among other miRNAs, miR-204-5p is down-regulated and its expression regulates EMT during Human PCO by Targeting SMAD4 as one of the mediators of TGF-β/SMAD signaling, as a predicted target of miR-204-5p (25).

According to these evidences and to test whether SMAD4 silencing decreases the gene expression level of hsa-miR-204, as one of the most downregulated miRNAs in SDCs, we selected miR-204 and detected its expression by Taq Man miRNA assay. As expected, miR204 levels were more than 18-fold lower in SDCs than in the corresponding PCs and were significantly upregulated in SDCs after transfection (*P <*0.0001) (**Figure 9D**).

To test whether SMAD4 silencing decreases the gene expression level of hsa-miR-204, as one of the most downregulated miRNAs in SDCs (7), we detected its expression by Taq Man miRNA assay.

## Discussion

Despite the advances in traditional therapies that aim to eliminate the bulk of the tumor, poor cure rates may result from the ability of cancer to repopulate and spread after initial therapies due to the presence of CSCs (26). The resistance of renal CSCs to chemotherapy and radiotherapy prepared the rationale for novel therapeutic strategies targeting this invasive cell population, inducting cell differentiation, and blocking CSCs maintenance pathways (27). Targeting these CSCs through EMT inducer pathways, such as TGFβ, Wnt, Notch and Hedgehog signaling pathways, may cause lasting and complete regression; this could enhance treatment outcomes for patients with RCC and improve prognosis (28).

In this study, we isolated SDCs from PCs in a medium that is commonly applied to support the growth of CSCs. These SDCs expressed significantly higher levels of stem cell-related transcription factors, increased expression of CSC marker, sphere-forming ability, and clonogenicity, larger colony size, and more invasive cells than the PCs, which are all CSC related features (29) and in agreement with the study by Lichner Z et al. (7).

TGFβ signaling is necessary for carcinoma metastasis, cell invasiveness, and angiogenesis during dedifferentiation in late-stage tumors (30). We provided multiple lines of evidence that the TGFβ-EMT axis is an active contributor to RCC sphere formation. Considering the strong links between EMT and invasiveness and the pivotal role of invasiveness in cancer progression and metastasis, our findings suggested that most of the mesenchymal genes were upregulated in our SDCs population. Moreover, for the first time, we observed a significant increase in SMAD4 and TGFβ-1 in SDCs compared to PCs.

In line with our results, another study revealed that SMAD4-expressing cells exhibited an increased TGFβ-induced EMT response (31). Gulubova M et al. conducted a study on the expression of signaling proteins SMAD4, SMAD7, and TGF. They found that the majority of colorectal cancers expressed SMAD4 and TGF-β1 and suggested that hypoxia-induced TGF-β1 production by tumor cells may suppress the tumor-infiltrating immune cells and contribute to their invasiveness through autocrine activation of Smad signaling (32). It has been shown that TGF-β1 gene expression and secretion can elevate tumor cell growth through the paracrine effects of the mesenchymal stem cells (MSCs) (33), which is matched with the increased TGF-β1 and EMT related genes in this study.

Furthermore, significant increase in VEGFA gene expression levels was observed in SDCs, which is a potent angiogenic factor and has been shown to drive malignant stem cells and also promotes breast and lung CSC self-renewal (34).

Also, the results of flow cytometric analysis exhibited that SDCs express the mesenchymal markers, which are compatible with the mesenchymal cell related surface marker expression reported in the self-renewing cell population of human embryonic renal cell line and renal cancer (35). Moreover, expression of putative CSC markers in isolated SDCs compared to PCs showed that these SDCs can be considered as a cancer stem-like cell population.

SMAD4 deletions or mutations have been widely observed in different cancer types, such as colorectal and pancreatic cancers (36). Loss of heterozygosity (LOH) has been reported in RCC patients (37). SMAD4 has been extensively studied and found to have a role in the tumorigenesis of many human cancers including gastric and colorectal cancers (38). In a research conducted by Zhao S et al, cells expressing SMAD4 exhibited an increased TGF-β-mediated EMT and the SMAD4 inhibition suppressed the TGF-β–mediated invasion and metastasis in pancreatic cancer cells (31).

In light of the pro metastasis and oncogenic role, many strategies are being applied to target the TGF-β pathway as a treatment for metastatic tumors, including RCC (39). Moreover Small and large molecules have been used as TGF-β pathway inhibitors in a variety of different tumor models in preclinical studies (39).

Although some studies have attempted to evaluate the role of SMAD4 and TGF-β signaling in RCCs (40), little has been uncovered on the associations between SMAD4 expression and its clinical significance to RCC subtypes and the detailed mechanism of SMAD4 inhibition in renal CSC.

This was the first study on the nuclear expression of SMAD4 in RCC subtypes in which the clinical significance and prognostic value of SMAD4 expression patterns were investigated. Nuclear expression of SMAD4 was accompanied with RCC subtypes and renal pelvis involvement. The ccRCC and papillary RCC subtypes had higher expression of SMAD4 than chRCC, which may be related to their more invasive tumor behavior. It has been revealed that ccRCC and pRCC samples have worse outcomes and higher potential to be aggressive and develop metastasis compared to chRCC (41).

In this study different RCC subtypes exhibited a statistically significant difference between the nuclear expression of SMAD4. However, we could not observe any association between the nuclear SMAD4 expression and higher nucleolar grade, tumor stage, and other clinicopathological findings in RCC samples, which is in line with the previous study by Cardillo et al. (42). In contrast, JH Park et al. found that low expression of SMAD4 was positively correlated with histological grade and PT Stage (43).

Our results indicated that nuclear SMAD4 expression and tumor size were independent significant risk factors affecting the DSS of patients with RCC and ccRCC as the most metastatic subtype in multivariable analysis. These results are in line with our previous research on the same set RCC TMA blocks which indicated that the cytoplasmic expression of Twist1, as an EMT-related transcription factor was associated with higher grades renal cell carcinomas and worse progression-free survival in clear cell renal cell carcinoma (24). Both of our researches indicate more evidence for the role of EMT in RCC pathogenies and its worse prognosis. Although Cox proportional analysis by JH Park et al. showed that low SMAD4 expression was significantly accompanied with progression-free survival, it was not significantly associated with DSS (43). The discrepancy between these 2 studies may be due to different reagents, such as a primary anti-SMAD4 antibodies, and occurrence of cancer-related deaths in RCC patients. Furthermore, unlike our study, they only used 1 core from each case and indicated that they could not overcome potential bias from tumor heterogeneity.

Our data, for the first time, showed that the inhibition of SMAD4 in PCs leads to a significant decrease in SMAD4 at both gene expression and protein levels in post transfected SDCs after SMAD4 inhibition, compared to SDCs before transfection with SiSMAD4. Furthermore, the inhibition of SMAD4 in PCs reduces the formation and/or stabilization of highly invasive SDCs with mesenchymal properties. Our findings are resemble to a previous study on glioblastoma stem cells which found SMAD inhibition may cause GBM stem cells to differentiate to CD133 cells and reduce their tumorigenicity (44).

In the present study, miR 204 level was more than 18-fold lower in SDCs than in the PCs and it is significantly decreased in post transfected SDCs. It has been suggested that miR-204 is one of the most downregulated miRNAs in SDCs. SMAD4 is predicted to be the direct target of miR-204 in TGF-β signaling pathway in SDCs and participate in EMT activation and stem cell-like properties (25). In a recent study, TGF-β2–induced EMT in the presence of SMAD4 small interfering RNA was inhibited due to miR-204-5p overexpression (7). Given the inverse relationship between SMAD4 and miR-204 in our study, miR-204 may have a role in SMAD4 inhibition in TGF-β signaling pathway and reduction in the EMT and stem cell properties in renal CSC; however, its contribution needs to be more investigated.

We examined the expression profiles of several stemness genes. Lin28, Nestin, and Rex1 showed significant lower expression in SDCs compared to the same population before SMAD4 inhibition. Several researchers have revealed that knockdown or inhibition of stemness genes, including Lin28, results in reduction of CSC characteristics (45). On the other hand, some experimental findings suggest that the capacity for self-renewal is related to Nestin expression (46). Taken together, our results suggest that targeting SMAD4 may reduce the stemness potential of the renal CSCs.

Moreover, we analyzed the expression of several EMT-related genes and observed decreased expression of Vimentin, Snail1, Snail2, Twist1, Twist2, N-cad, in SDCs post transfection compared to SDCs. It has been shown that expression of Twist1 and Twist2 as mesenchymal genes has been accompanied with tumor progression in human solid tumors (47) and the inhibition of snail1 in mesenchymal cells can lead to loss of self-renewal characteristics *in vitro* through downregulation of Nanog (48). Our previous research about the association of the cytoplasmic expression of Twist1, in worse progression-free survival of ccRCC (24), validates our *in vitro* finding about post transfection reduction of Twist1 in SDCs as one of the EMT targets and provides more evidences for the role of EMT in renal cancer pathogenesis.

Considering the strong links between invasiveness and EMT and the fundamental role of invasiveness in metastasis of cancer cells, our findings suggest that reducing these genes in our SDCs population after SMAD4 inhibition may offer new therapeutic approaches for RCC.

Our findings showed that cancer cells that grew post transfection (SDCs) had a significantly lower sphere-forming ability and invasive potential and CXCR4, CD133, and CD105 markers were less expressed compared to pre transfection. These markers have been known as renal CSC marker in previous studies; therefore, we suggest that SMAD4 inhibition reduces the stemness properties of cancer cells. M Gassenmaier et al. found that CXCR4 seems to be one of the most prevalent CSC markers in solid tumor and is required for maintenance of RCC initiating cells and can predict metastasis at the time of diagnosis (49). A previous study found that CD105 expressing subpopulation in human RCC xenograft and patient samples have a greater capacity to form spheres *in vitro* and its knockdown can reduce sphere-forming ability and tumorigenicity (50). In another study CD133 was examined as an identifying marker for CSCs in renal carcinomas and found to have a role in tumor angiogenesis (51).

TGF- β signaling pathway has a central role in mesenchymal cell maintenance and the TGFβ regulated EMT in stem cell renewal (7). In the present study, increased expression of mesenchymal markers and decreased expression of epithelial markers, such as CD34 and CD24, were observed in post transfection SDCs compared to SDCs before transfection. In our study, some of the gene expressions and the surface markers were not in parallel with our hypothesis, indicating a decreased EMT-related property in transfected SDCs. However, considering all results, including sphere-forming, clonogenicity potential, and invasiveness of cancer cells, our data suggest that SMAD4 inhibition reduces EMT‐induced renal CSC properties.

To conclude, we isolated SDCs from a metastatic cell line and found that they present cancer stem cell/mesenchymal properties, including the formation of self-renewing SDCs in serum free defined media, increased invasiveness, high clonogenicity, increased expression levels of stemness and EMT-related genes and increased expression of putative CSCs markers.

We also presented evidence that the activated TGFβ-EMT axis, including elevated SMAD4 and TGFβ1 gene expressions, has a role in gaining the EMT-related properties in SDCs from a metastatic cell line model. This was mainly based on the exploring that the inhibition of SMAD4 simultaneously with the decreased TGFβ1 reduces the formation of highly invasive cancer spheres in PCs, decreases clonogenicity, and invasiveness of cancer cells and leads to increase in EMT-related properties. We also found that increased SMAD4 predicts prognosis and there is a significant correlation with SMAD4 and survival outcomes in RCC patients and ccRCC subtype as the most metastatic RCC subtype.

Our findings, for the first time, propose that targeting SMAD4 as a pivotal transducer of the TGF-β-signaling pathway may be effective as a supplementary targeted therapy against renal CSCs and may improve the RCC prognosis, particularly the ccRCC subtype. However, the functional role of Smad proteins and their contribution to TGF-β/Smad signaling pathway, as the major deriving force for EMT in regulation of tumor-initiating potential of renal CSCs and stem cell properties, is yet to be defined.

## Data Availability Statement

The raw data supporting the conclusions of this article will be made available by the authors, without undue reservation.

## Ethics Statement

All procedures involving human participants were done in accordance with the ethical standards of the institutional and/or national research committee and with the 1964 Helsinki declaration or its later amendments or comparable ethical standards. This study was approved by Iran University of Medical Sciences Research Ethics Committee (number: 25166). The patients/participants provided their written informed consent to participate in this study. Written informed consent was obtained from the individual(s) for the publication of any potentially identifiable images or data included in this article.

## Author Contributions

AR performed all laboratory procedures and data analysis, and wrote the primary manuscript supervised by MM, ZM, and SB who designed this research study. SB and MAb were consultants in this project. LSZ contributed to most of the experiments in the lab. All authors were involved in the review of the manuscript and approved the final version of this manuscript.

## Funding

This study was a part of a PhD thesis and supported by a grant from Iran University of Medical Sciences (Grant #25166). The authors would like to express their gratitude for financial support from Iran National Science Foundation (INSF) (number: 94009372).

## Conflict of Interest

The authors declare that the research was conducted in the absence of any commercial or financial relationships that could be construed as a potential conflict of interest.
