# Supplementary material for: SMAD4 Expression in Renal Cell Carcinomas Correlates With a Stem-Cell Phenotype and Poor Clinical Outcomes
Source: Front Oncol. 2021 May 3;11:581172. doi: 10.3389/fonc.2021.581172 (PMC8127783; doi:10.3389/fonc.2021.581172)
Supplement: Supplementary file 1 [file Table_1.pdf]

The PCR primers are listed in supporting information Supplementary Table 2 (Table S2).

Table S2. Primers sequences for quantitative Real-time PCR (qRT-PCR)

**Genes groups**	**Gene name**	**Primer Sequence (5´→ 3´)**	**Product size base pair(bp)**
Housekeeping gene	GAPDH	F: CATGAGAAGTATGACAACAGCCT<br>R: AGTCCTTCCACGATACCAAAGT	113
Stemness genes	OCT4	F: GTGGAGAGCAACTCCGATG<br>R:TGCAGAGCTTTGATGTCCTG	121
	SOX2	F: AATGGGAGGGGTGCAAAAGAGG<br>R: GTGAGTGTGGATGGGATTGGTG	143
	Nanog	F: AGCTACAAACAGGTGAAGAC<br>R: GGTGGTAGGAAGAGTAAAGG	145
	Klf4	F: CCTCGCCTTACACATGAAGAG<br>R: CATCGGGAAGACAGTGTGAAA	108
	C-MYC	F: ACACATCAGCACAACTACG<br>R: CGCCTCTTGACATTCTCC	161
	Lin28	F: CTTTGCCTTCGGACTTCTC<br>R: AACTGCTGGTTGGACACG	100
	Nestin	F: AGAGAGCGTAGAGGCAGTAA<br>R: GGTGCTTGAGTTTCTGGAGAT	108
	Rex1	F: TTTACGTTTGGGAGGAGG<br>R: GTGGTCAGCTATTCAGGAG	150
EMT genes	Snail1	F: CCAGAGTTTACCTTCCAGCA<br>R: GATGAGCATTGGCAGCGA	101
	Snail2	F: AACTACAGCGAACTGGACAC<br>R: GGATCTCTGGTTGTGGTATGAC	91
	E-cadherin	F: CAGGAGTCATCAGTGTGGT<br>R: GGAGGATTATCGTTGGTGTCAG	151
	N-cadherin	F: GCCCAAGACAAAGAGACCC<br>R: CTGCTGACTCCTTCACTGAC	94

	Twist1	F: TTCTCGGTCTGGAGGATGGAG<br>R: ACGCCCTGTTTCTTTGAATTTGG	228
	Twist2	F: AGCGACGAGATGGACAATAAG<br>R: TAGTGGGAGGCGGACAT	116
	Zeb1	F: CTTCTCACACTCTGGGTCTTATTC<br>R: CGTTCTTCCGCTTCTCTCTTAC	75
	Zeb2	F: GAGAAAGTACCAGCGGAAACA<br>R: AGGAGTCGGAGTCTGTCATATC	98
	Vimentin	F: TCTACGAGGAGGAGATGCGG<br>R: GGTCAAGACGTGCCAGAGAC	213
Angiogenesis genes	VEGFA	F: GCTTACTCTCACCTGCTTCTG<br>R: CTGTCATGGGCTGCTTCTT	97
	EGF	F: CTTGTCATGCTGCTCCTCCTG<br>R: TGCGACTCCTCACATCTCTGC	118
TGFβ1	TGFβ1	F: AACCCACAACGAAATCTATGAC<br>R: TAACTTGAGCCTCAGCAGAC	142
ABC transporter genes	ABCG2	F: TTCCACGATATGGATTTACGG<br>R: GTTTCCTGTTGCATTGAGTCC	83
	ABCB1	F: GTTCAGGTGGCTCTGGATAAG<br>R: AGCGATGACGTCAGCATTAC	93
	ABCC1	F: CGCCTTCGCTGAGTTCCT<br>R: TGCGGTGCTGTTGTGGTG	217
SMAD4	SMAD4	F: TGATGACCTTCGTCGCTTAT<br>R: TGATGCTCTGTCTTGGGTAATC	148
miR-204	miR-204	-	-
