# Supplementary material for: SMAD4 Expression in Renal Cell Carcinomas Correlates With a Stem-Cell Phenotype and Poor Clinical Outcomes
Source: Front Oncol. 2021 May 3;11:581172. doi: 10.3389/fonc.2021.581172 (PMC8127783; doi:10.3389/fonc.2021.581172)
Supplement: Supplementary file 2 [file Table_2.pdf]

**Table S3.** The association between nuclear SMAD4 expression and clinicopathological parameters in three subtypes of RCC

| | | Expression of SMAD4 | | |
|---|---|---|---|---|
| | | Nuclear *H-Score* (N %) | | * *P value* |
| **Patient and tumor charachteristics** | **Total No.Cases 209(N %)** | **Low** (≤200) | ***High*** (200<) | |
| **Age (y)** | | | | |
| ≤55.0 | 102(48.8) | 93(44.5) | 9(4.3) | **0.046** |
| >55.0 | 107(51.2) | 87(41.6) | 20(9.6) | |
| **Sex** | | | | |
| Male | 180(67.5) | 120(57.4) | 60(28.7) | 0.671 |
| Female | 29(32.5) | 21(10.0) | 8(3.8) | |
| **RCC subtypes** | | | | |
| ccRCC | 158(75.6) | 132(63.2) | 26(12.4) | |
| ChRCC | 17(8.1) | 17(8.1) | 0(0.0) | **0.034** |
| pRCC | 34(16.3) | 31(14.8) | 3(1.4) | |
| **Tumor size(cm)** | | | | |
| <4 | 43(20.6) | 33(15.8) | 10(4.8) | |
| 4-7 | 72(34.4) | 65(31.3) | 7(3.3) | 0.155 |
| 7-10 | 50(23.9) | 42(20.1) | 8(3.8) | |
| >10 | 44(21.1) | 40(19.1) | 4(1.9) | |
| **Tumor Stage** | | | | |
| I/II | 85(81.8) | 71(34.0) | 14(6.7) | 0.369 |
| III/IV | 124(18.2) | 109(52.2) | 15(7.2) | |
| **Nuclear Grade** | | | | |
| I/II | 116(60.4) | 97(50.5) | 19(9.9) | 0.542 |
| III/IV | 76 (39.6) | 66(34.4) | 10(5.2) | |
| **Renal pelvis involvement** | | | | |
| Yes | 188(90.0) | 165(78.9) | 23(11.0) | **0.04** |
| No | 21(10.0) | 15(7.2) | 6(2.9) | |

| | | | | |
|---|---|---|---|---|
| | | | | |

* Significances are based on Pearson  Chi-square test

Values in bold are statistically significant.

ccRCC indicates clear cell Renal Cell Carcinoma; ChRCC, chromophob Renal Cell Carcinoma and pRCC, papillary Renal Cell Carcinoma

H-score, histological score
