# Supplementary material for: SMAD4 Expression in Renal Cell Carcinomas Correlates With a Stem-Cell Phenotype and Poor Clinical Outcomes
Source: Front Oncol. 2021 May 3;11:581172. doi: 10.3389/fonc.2021.581172 (PMC8127783; doi:10.3389/fonc.2021.581172)
Supplement: Supplementary file 3 [file Table_3.pdf]

Table S4. Survival table of patients with RCC (Renal Cell Carcinoma) and ccRCC (clear cell Renal Cell Carcinoma).

	Year	1	2	3	4	5	6	7	8	9
RCC patients	Low nuclear SMAD4 expression (N=100)									
	N of cumulative Events (censored)	10	12	13	13	13	13	16	14	14
	N.at risk	88	76	64	52	40	28	14	4	0
	High nuclear SMAD4 expression (N=24)									
	N of cumulative Events (censored)	7	8	-	-	-	-	-	-	-
	N.at risk	12	0							
ccRCC patients	Low nuclear SMAD4 expression (N=76)									
	N of cumulative Events (censored)	8	9	9	9	10	10	10	-	-
	N.at risk	64	52	40	28	16	4	0	-	-
	High nuclear SMAD4 expression (N=22)									
	N of cumulative Events (censored)	7	8	-	-	-	-	-	-	-
	N.at risk	10	0	-	-	-	-	-	-	-
