# Supplementary material for: SMAD4 Expression in Renal Cell Carcinomas Correlates With a Stem-Cell Phenotype and Poor Clinical Outcomes
Source: Front Oncol. 2021 May 3;11:581172. doi: 10.3389/fonc.2021.581172 (PMC8127783; doi:10.3389/fonc.2021.581172)
Supplement: Supplementary file 4 [file Table_4.pdf]

Table S5. Comparison of clonogenicity and size of colony in parental cells (PCs) compared spheroid-derived cells (SDCs) before and after transfection

Colony types	Mean number of cells±SD			*P* value	Mean colony size (μm)±SD			*P* value
	PCs	SDCs before transfection	SDCs after transfection		PCs	SDCs before transfection	SDCs after transfection	
Holoclone	36.6 ±10.2	73.0 ±4.5	28.6 ±5.1	*<0.05*	1793.3 ±130	4501.6 ±172	1578.3 ±82.0	*<0.05*
Meroclone	32.3 ±3.5	45.3 ±6.1	22.3 ±3.21		1233.3 ±48.8	2852.6 ±41.5	1180.3 ±76.7	
Paraclone	16.3 ±0.5	34.0 ±1.7	15.6 ±6.4		884.6 ±71.1	1695.3 ±11.1	760.0 ±73.2	

SD: Standard deviation
