# Supplementary material for: SMAD4 Expression in Renal Cell Carcinomas Correlates With a Stem-Cell Phenotype and Poor Clinical Outcomes
Source: Front Oncol. 2021 May 3;11:581172. doi: 10.3389/fonc.2021.581172 (PMC8127783; doi:10.3389/fonc.2021.581172)
Supplement: Supplementary file 5 [file DataSheet_1.pdf]

Supplementary Appendix, S1.

The standard curve was plotted for cell invasion assay

We performed a cell invasion assay to evaluate the invasive properties of PCs and SDCs. Using the manufacturer's recommendations, a standard curve was generated and best-fit line plotted from the point zero based on the linear equation and regression coefficient or R2 (Coefficient of determination). The average of all wells for each condition was calculated and background was subtracted from averages. The trend line equation was used to determine the number of cells present in each well; for the equation, y = mx + b, y value was replaced with relative fluorescence units (RFU), and solved for X (number of cells per well). The data used for plotting the standard curve is provided in the following supplemental table1 (Table S1) and Figure S1 shows the standard curve.

Table S1: The data used for plotting the standard curve

Cells/Well	Average RFU*	Background	Corrected RFU
50000	15663	-236	15427
25000	8434	-236	8198
10000	4634	-236	4398
5000	2448	-236	2212
2500	1412	-236	1176
1000	722	-236	486
0	236		

* RFU indicates Relative Fluorescence Units

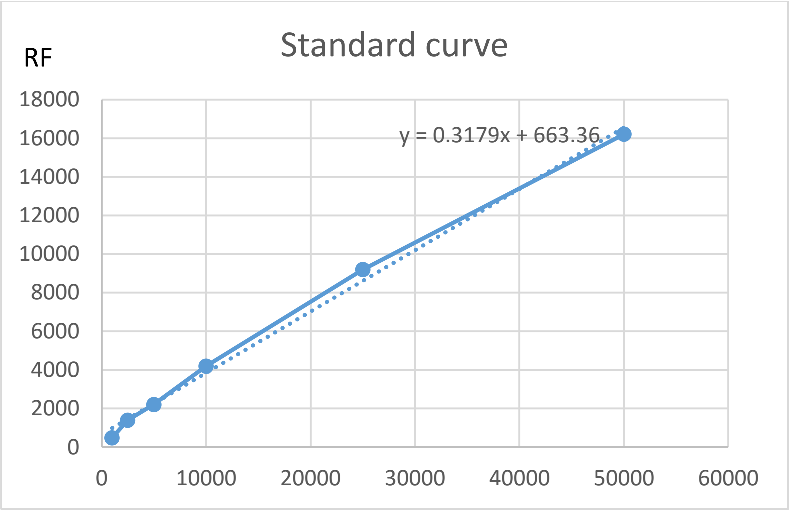

FigureS1. The graph shows a cell invasion standard curve. ACHN cells were harvested, diluted, incubated for one hour with Calcein-AM, and assayed for fluorescence. The trend line and line equation were included on the graph.
