# Supplementary material for: SMAD4 Expression in Renal Cell Carcinomas Correlates With a Stem-Cell Phenotype and Poor Clinical Outcomes
Source: Front Oncol. 2021 May 3;11:581172. doi: 10.3389/fonc.2021.581172 (PMC8127783; doi:10.3389/fonc.2021.581172)
Supplement: Supplementary file 6 [file DataSheet_2.pdf]

Supplementary file S5. cytoplasmic expression of Twist1, as an EMT-related transcription factor was associated with higher grades renal cell carcinomas and worse progression-free survival in clear cell renal cell carcinoma.

Immunohistochemical (IHC) analysis of Twist1 expression in different renal cell carcinoma (RCC) samples:

The cytoplasmic and nuclear expression of Twist1 was examined in 252 well-defined renal tumor tissues, including 173 (68.7%) clear cell renal cell carcinomas (ccRCC), 45(17.9%) papillary renal cell carcinomas (pRCC) and 34 (13.5%) chromophobe renal cell carcinomas (ChRCC), by immunohistochemistry on a tissue microarray (TMA). The association between expression of this marker and clinicopathologic parameters and survival outcomes were then analyzed.

RCC tissue samples were collected from the Hasheminejad Kidney Center, a major university-based referral Urology-Nephrology center in Tehran, Iran, from 2007 to 2015. Patients who had undergone radical nephrectomy and who had no history of preoperative hormone or radiation therapy were included in the current study. On the basis of the pathology findings and case records, the patient samples were categorized into 3 groups: ccRCC (n=173), pRCC (n=45), and ChRCC (n=34). The specimens were embedded in paraffin using a routine pathologic tissue processing technique. Medical records were retrieved to obtain clinicopathologic parameters including, tumor size, metastasis to regional lymph node & renal vein (RVI) and microvasulcar invasions (MVI) and also the Gerota's fascia, adrenal gland, peripheral fat, and renal pelvis involvements. In addition, pathologic tumor stage was defined according to the pTNM Classification for Renal Cell Carcinoma. All data of patients were kept fully de-identified in all steps. This research study was approved by the Iran University of Medical Sciences Research Ethics Committee.

Construction of tissue microarrays (TMAs) and immunohistochemistry (IHC) staining

The renal tissue TMAs was prepared and constructed in three copies, each containing one sample from a different region of the tumor. IHC staining was performed according to a standard chain polymer-conjugated (Envision) technique. Briefly, sequential TMA sections were dewaxed (60ºC for 20 min) and rehydrated in xylenes, followed by graded ethanol treatment. Antigen was retrieved by autoclaving tissue sections for 10 minutes in sodium citrate buffer (pH 6.0). Endogenous peroxidase and nonreactive staining were blocked by 3% H2O2 for 20 minutes at room temperature. The sections were then incubated overnight at 4ºC with rabbit monoclonal antibody against Twist1 (ab49254; Abcam, UK) using a 1:100 dilution.After three washes in Tris-buffered saline (TBS), sections were incubated with anti-rabbit/anti-mouse Envision (Dako, Denmark) as the secondary antibody for 15 minutes. TMA slides were treated with 3, 3´-diaminobenzidine (DAB, Dako) substrate as a chromogen for 10 minutes at room temperature. Sections were lightly counterstained with hematoxylin, dehydrated in alcohol, cleared with xylenes, and mounted. For negative control, the primary antibody step was replaced with TBS and only the secondary antibody was used. Human testis tissue were used as a positive control for Twist1 staining.

Evaluation of immunostaining

The immunostained tissue arrays were examined using a semi-quantitative scoring system by two investigators (MA and AR) in a coded manner without previous knowledge of clinical and pathological parameters of patients. In difficult cases, the scoring was confirmed by two observers and a consensus was achieved.

Scoring system

The Twist1 staining of tissue sections was scored on a scales 0(absent), 1(weak), 2(moderate) or 3(strong) without previous knowledge of clinical and pathologic parameters. Immunostaining of Twist1 was performed.The overall score was obtained by H-score (Histochemical score) for each case by multiplying the intensity of staining by the percentage of positive cells and a final score of 0 to 300 was given to each core. The nuclear and cytoplasmic H-scores were classified into three groups: 0 -100 as group1 (low expression), 101 -200 as group 2 (moderate expression), and 201 -300 as group 3 (high expression).

Patient Characteristics

Of the 252 RCC samples that were included in the present study, 173 (68.7%) were ccRCC, 45 (17.9%) were pRCC, and 34 (18.5%) were ChRCC. One hundred and seventy-two (68.7%) samples were from male and 79 (31.3%) were from female patients. Overall, the mean age of the population was 55 (SD=13.1) years (25–82). Tumor size was categorized into four groups: ≤4 cm, 4–7 cm, 7–10 cm, and≥10 cm. (Turun et al. 2012) The median tumor size was 7 cm (5, 10), (1–21cm). Seventy-five (30.8%) specimens were stage I, 25(10.3%) were stage II, 134 (52.2%) were stage III and 18 (6.7%) were classified as stage IV. Ten (4.0%) specimens, had a low-nuclear grade (grade I), 118 (46.8%) were grade II, 77 (30.6%) were grade III and 13(5.2%) were classified as high-nuclear grade (grade IV). Regional lymph node involvement was found in 11 cases (6.4%), whereas 154 cases (89.0%) had no regional lymph node involvement and in 8 cases (4.6%) No lymph node was dissected during surgery. Thirty-five cases (18.7%) had MVI and 97cases (56.1%) had renal sinus invasion. Seventy-two cases (41.6%) had tumoral necrosis. Other reported involvements were: renal vein, 17 cases (6.7%); adrenal gland, 7 cases (4.0%); Gerota's fascia, 4cases (2.3%); renal pelvis, 13 cases (7.5%), renal sinus 135 cases (4.9.6%); and peripheral fat, 32cases (18.5%).

Comparison of Twist1 expression in RCC subtypes

Analysis of TMA-based IHC staining demonstrated that expression of Twist1 was localized to the cytoplasm and nucleus of tumor cells. In RCC, the respective cytoplasmic and nuclear expression rate for Twist1 was 98.8% (249/252) and 91.3% (231/252), with varying levels of intensities.

The level of expression was examined by three scoring methods: intensity of the staining, percentage of Twist1-positive tumor cells, and H-score. Twist1 expression in RCC subtypes is illustrated in Fig.1 and Table 1.

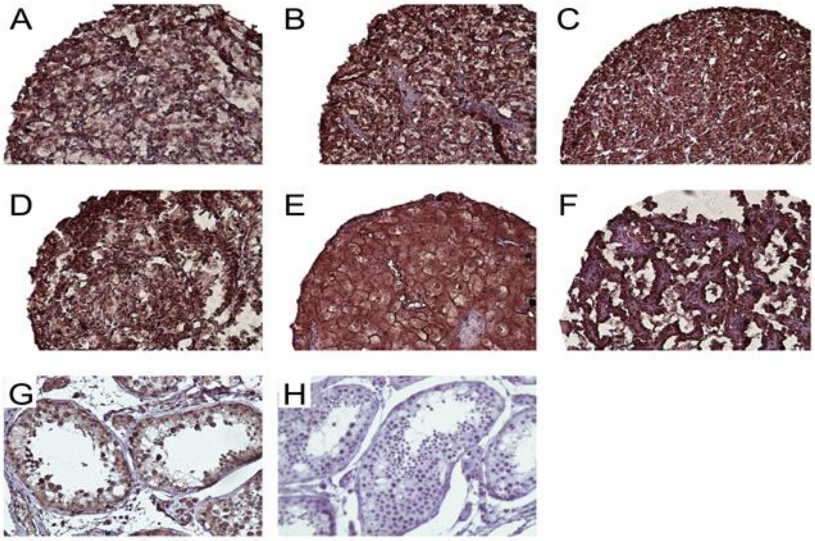

Fig 1. Immunohistochemical (IHC) analysis of Twist1 expression in different renal cell carcinoma (RCC) samples. RCC samples expressed Twist1 at various levels. Cytoplasmic expression of Twist1 in clear cell RCC at various levels: weak (A), moderate (B), and strong (C), chromophobe RCC (strong) (E) and papillary RCC (moderate) (F). Strong nuclear expression of Twist1 in clear cell RCC (D). IHC staining of normal testis tissue as positive (G) and negative (H) controls. Figures are shown with a magnification of 200×).

The nuclear and cytoplasmic expressions of Twist1 in each subtype of RCC are presented in Table 1.

Table 1. Twist1 expression (Intensity, Percentage of positive tumor cells, and H-Score) in ccRCC, chRCC and pRCC tissues.

Twist1 Expression (Scoring System)	Nuclear				Cytoplasmic			
**RCC subtypes**	**ccRCC N (%)**	**ChRCC N (%)**	**pRCC N (%)**	***P value**	**ccRCC N(%)**	**ChRCC N (%)**	**pRCC N(%)**	***P value**
**Intensity of staining**								
Negative	7(0.4)	1(0.6)	1(2.9)		9(21.4)	1(2.2)	0(0.0)	
Weak	88(50.9)	62(35.8)	30(88.2)	**<0.001**	24(57.1)	11(24.4)	16(47.1)	0.426
Intermediate	76(43.9)	78(45.1)	3(8.8)		8(19.0)	25(55.6)	13(38.2)	
Strong	2(1.2)	32(18.5)	0(0.0)		1(2.4)	8(17.8)	5(14.7)	
**Percentage of positive tumor cells**								
<25								
25-50	30(17.3)	10(29.4)	18(42.9)		6(3.5)	0(0.0)	1(2.2)	
50-75	53(30.6)	12(35.3)	8(19.0)	***0.005***	6(3.5)	1(2.9)	1(2.2)	0.822
75<	32(18.5)	7(20.6)	1(2.9)		4(2.3)	2(5.9)	3(7.1)	
	58(33.5)	5(14.7)	13(31.0)		157(90.8)	31(91.2)	41(91.1)	

***H*-Score**								
Low (0-100)	117(67.6)	31(91.2)	36(85.7)		63(36.4)	17(50.0)	13(28.9)	
Moderate (101-200)	54(31.2)	3(8.8)	5(11.9)	***0.011***	78(45.1)	12(35.3)	24(53.3)	0.406
High (201- 300)	2(1.2)	0(0.0)	1(2.4)		32(18.5)	5(14.7)	8(17.8)	
**Total**	173	34	45		173	34	45	

* Significances are based on Pearson Chi-square test
Values in bold are statistically significant.
ccRCC indicates clear cell Renal Cell Carcinoma; ChRCC, chromophob Renal Cell Carcinoma;
pRCC, papillary Renal Cell Carcinoma

The difference between the nuclear expression of Twist1 between ccRCC and ChRCC subtypes and also ccRCC and pRCC subtypes was statistically significant. (Fig. 2) The difference between the cytoplasmic expression of Twist1 in the different RCC subtypes was not statistically significant (P = 0.276).

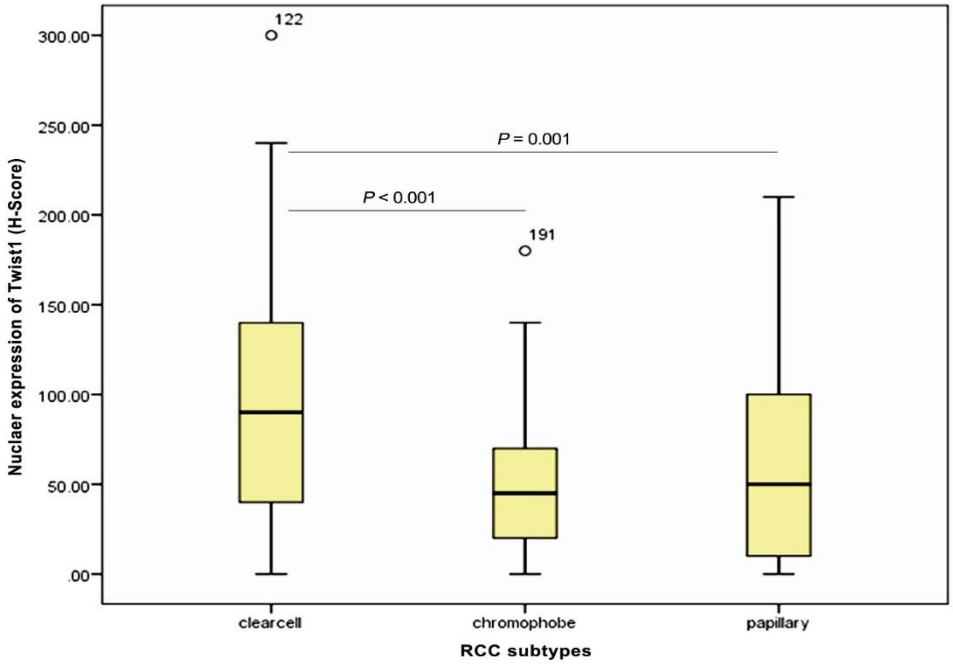

Fig 2. Analysis of Twist1 nuclear expression levels in different renal cell carcinoma (RCC) tissues, including clear cell RCC (ccRCC), chromophobe RCC (ChRCC), and papillary RCC (pRCC) using Mann-Whitney U test. On the basis of the standard definitions, the box-plot shows the median (bold line), interquartile lines (box), and outlier observation (circle).

Association of Twist1 nuclear and cytoplasmic expression with clinicopathologic parameters in RCC
The difference between the median cytoplasmic expression of Twist1 in the low (I & II) and high (III & IV) grades RCC groups was significant (P = 0.046). The cytoplasmic expression of Twist1 was significantly associated with tumor size, RVI, and MVI. (P values were 0.012, 0.031, and 0.044, respectively) (Table 2).

Table 2. Association of Nuclear and Cytoplasmic Twist1 expression (*H*-Score) and clinical characteristics in RCC patients.

		**Expression of Twist1**							
		**Nuclear *H-Score* (N %)**			**** P value***	**Cytoplasmic *H-Score* (N %)**			**** P value***
**Patient and Tumor Charachteristics**	**Total No.Cases 252 (N %)**	**Low (0-100)**	**Moderate (101-200)**	***High* (201-300)**		**Low (0-100)**	**Moderate (101-200)**	***High* (201-300)**	

**Age (y)**<br>≤55<br>>55	 124(49.2)<br>128(50.8)	 49(39.5)<br>44(34.4)	 53(42.7)<br>61(47.7)	 22(17.7)<br>23(18.0)	0.120	 49(39.5)<br>44(34.4)	 53(42.7)<br>61(47.7)	 22(17.7)<br>23(18.0)	0.674
**Tumor size(cm)**<br><4<br>4-7<br>7-10<br>>10	 46(18.5)<br>92(36.9)<br>35(61.4)<br>57(22.9)	 35(76.1)<br>73(79.3)<br>35(61.4)<br>41(75.9)	 11(33.9)<br>19(20.7)<br>20(35.1)<br>12(22.2)	 0(0.0)<br>0(0.0)<br>2(3.5)<br>1(1.9)	0.162	 15(31.9)<br>43(46.7)<br>23(40.4)<br>12(21.4)	 22(46.8)<br>41(46.4)<br>24(42.1)<br>27(48.2)	 10(21.3)<br>8(8.7)<br>10(17.5)<br>17(30.4)	**0.012**
**Primary tumor (PT) Stage**<br>I/II<br>III/IV	 100(41.1)<br>152(58.9)	 74(74.0)<br>111(74.0)	 24(2.0)<br>38(25.2)	 2(2.0)<br>2(0.8)	0.626	 41(41.0)<br>52(34.2)	 45(45.0)<br>69(45.4)	 14(14.0)<br>31(20.4)	0.344
**Histological Grade**<br>I/II<br>III/IV	 128(50.7)<br>90(35.8)	 88(69.8)<br>65(73.0)	 36(28.6)<br>23(25.8)	 2(1.6)<br>1(1.1)	0.862	 52(40.6)<br>24(26.7)	 56(44.4)<br>43(48.3)	 18(14.1)<br>22(24.4)	***0.045***
**Renal vein invasion**<br>Present<br>Absent	17(6.7)<br>225(89.2)	7(41.2)<br>170(75.5)	9(52.9)<br>53(23.9)	1(5.9)<br>2(0.9)	**0.005**	3(17.6)<br>87(38.7)	7(41.2)<br>100(44.4)	7(41.2)<br>38(16.9)	***0.031***
**Microvascular invasion**<br>Present<br>Absent	47(18.7)<br>182(72.2)	32(68.1)<br>136(74.7)	15(31.9)<br>44(24.2)	0(0.0)<br>2(1.1)	0.652	12(25.5)<br>73(39.5)	20(42.6)<br>84(45.4)	15(31.9)<br>28(15.1)	***0.044***

* Significances are based on Pearson Chi-square test
Values in bold are statistically significant.
ccRCC indicates clear cell Renal Cell Carcinoma; ChRCC, chromophob Renal Cell Carcinoma and pRCC, papillary Renal Cell Carcinoma

Analysis of Twist1 expression in each subtype

In order to compare RCC subtypes, we performed all analyses in RCC subtypes separately. The main results were as follows:

ccRCC (173samples)

A significant difference between the cytoplasmic expression of Twist1 in different grades (I/II Vs III/IV) was observed (P = 0.040). A significant correlation was reported between cytoplasmic Twist1 expression and grade (P = 0.026). A significant relationship was reported between nuclear Twist1 expression and lymph node involvement (P = 0.017).

pRCC (35 samples)

A significant difference was observed between the cytoplasmic expression of Twist1 in different stages (I/II Vs III/IV) (P = 0.036).

ChRCC (34 samples)

No significant associations were found between cytoplasmic and nuclear expression of Twist1 and clinicopathologic parameters.

Association of Twist1 expression with survival outcomes in RCC tissues

Of the 252 RCC samples that were included in the present study, 172 (52.4%) patients had no history of recurrence, metastasis, or disease related death. One hundred and twenty (47.6%) of the patients had the history of recurrence, metastasis, or disease related death. Forty-five (17.9%) patients had history of metastasis, while recurrence occurred only in 6 patients (2.4%). During follow-up time, disease-related death occurred in 39 patients (15.5%). The mean duration of follow-up time was 46.4 months (SD = 26.1), median was 42.5 months (29, 64), and range was 1–116 months. To further investigate the clinical usefulness of Twist1 expression in RCC, we compared DSS and PFS based on Twist1 expression.

Survival outcomes based on cytoplasmic Twist1 expression

The mean DSS time for patients with high, moderate, and low cytoplasmic expression of Twist1 was 64.4 (SD = 6.5), 82.6 (SD = 3.6), and 97.3(SD = 4.9) months, respectively. The 5-year DSS for patients whose specimens expressed high, moderate, and low cytoplasmic expression of Twist1 was 69.0, 78.0, and 84.0% respectively.

The mean PFS time for patients with high, moderate, and low cytoplasmic expression of Twist1 was 49.5 (SD = 3.8), 55.8 (SD = 2.7), and 59.6 (SD = 3.7) months, respectively. The 5-year PFS for patients whose specimens expressed high, moderate, and low cytoplasmic

levels of Twist1 was 23.0, 43.0, and 37.0 %, respectively (P = 0.294). The survival curves according to cytoplasmic Twist1 expression are depicted in Fig. 3

a

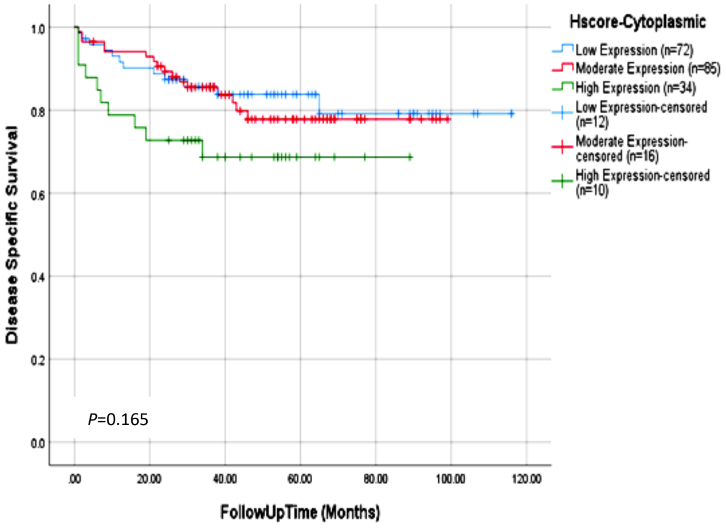

Number at risk by time

		Year	1	2	3	4	5	6	7	8	9
	Low Cytoplasmic Twist1 expression (N=72)										
		N of cumulative Events (censored)	9	10	11	11	12	10	12	-	-
		N.at risk	60	48	36	24	12	4	0	-	-
RCC patients	Moderate Cytoplasmic Twist1 expression (N=85)										
		N of cumulative Events (censored)	9	12	12	16	16	16	16	-	-
		N.at risk	73	61	49	37	25	13	1	-	-
	High Cytoplasmic Twist1 expression (N=34)										
		N of cumulative Events (censored)	9	10	10	-	-	-	-	-	-
		N.at risk	21	9	0	-	-	-	-	-	-

b

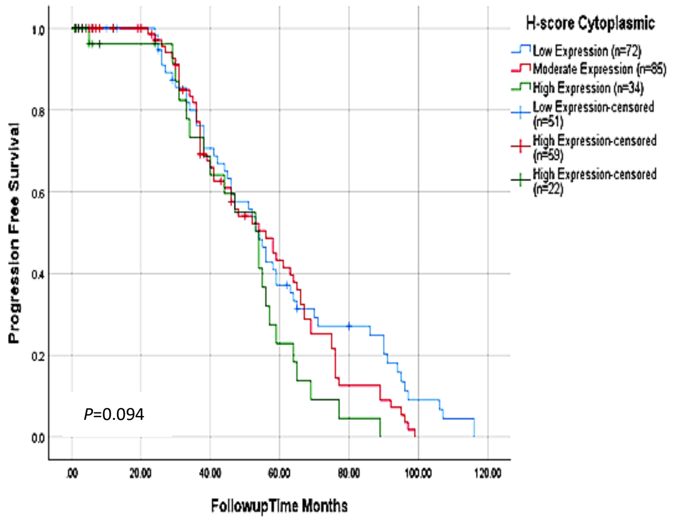

Number at risk by time

		Year	1	2	3	4	5	6	7	8	9
	Low Cytoplasmic Twist1 expression (N=72)										
		N of cumulative Events (censored)	0	7	18	30	39	51	-	-	-
		N.at risk	60	48	36	24	12	0	-	-	-
RCC patients	Moderate Cytoplasmic Twist1 expression (N=85)										
		N of cumulative Events(censored)	0	6	15	24	34	46	58	-	-
		N.at risk	73	61	49	37	25	13	1	-	-
	High Cytoplasmic Twist1 expression (N=34)										
		N of cumulative Events(censored)	1	12	22	-	-	-	-	-	-
		N.at risk	22	10	0	-	-	-	-	-	-

Figure 3: Correlation between cytoplasmic Twist1 expression and survival rates in patients with renal cell carcinoma. (A) Disease-specific survival (DSS) with cytoplasmic Twist1 expression. (B) Progression-free survival (PFS) with cytoplasmic Twist1 expression (cytoplasmic expressions were grouped into low- versus moderate- versus high expression levels).

Survival outcomes based on nuclear Twist1 expression

The 5-year DSS for the specimens which expressed high moderate and low nuclear levels of Twist1 was 78.0 and 80.0 %, respectively (P = 0.650); and for those expressing low nuclear levels of Twist1 was not computable due to the limited number of cases. Similarly, the 5-year PFS for the specimens expressing high and moderate levels of Twist1 was 35, 45 % respectively, and non-computable for low nuclear levels of Twist1 (P = 0.611). Due to the low number of remaining cases in the group with high nuclear expression of Twist1, survival cures were not exploited.

To investigate whether Twist1 expression was an independent prognostic predictor of DSS and PFS, and to assess the clinical significance of various parameters that might influence survival outcomes, univariate and multivariable analyses were performed. As shown in Table 4, clinical stage (P = 0.002), Fuhrman nuclear grade (P < 0.001), and tumor size (P < 0.001) were significant risk factors affecting the DSS of patients with RCC, but cytoplasmic Twist1 expression (P = 0.179), was not a significant risk factor in univariate analysis. Clinical stage, Fuhrman nuclear grade, and tumor size remained significant risk factors in multivariable analysis (P values 0.031, 0.003, and 0.018, respectively).

Clinical stage of RCC was a risk factor for PFS in univariate and multivariable analysis (*P* = 0.027). No risk factor was found for PFS in multivariable analysis. Other clinicopathologic variables including metastasis to regional lymph node & RVI, MVI; and also the Gerota's fascia, adrenal gland, peripheral fat, and renal pelvis involvements, were not significant factors affecting the DSS and PFS of patients with renal cancer.

Table 3. Univariate and multivariable analysis of disease free survival (DSS) and progression-free survival (PFS) in patients with renal cell carcinoma (RCC).

**Feature**	**DSS**				**PFS**			
	**Univariate**		**Multiivariable**		**Univariate**		**Multiivariable**	
	**HR (95%CI)**	***P* value**	**HR (95%CI)**	***P* value**	**HR (95%CI)**	***P* value**	**HR (95%CI)**	***P* value**
**Age**								
(>55 vs.≤55)	1.4(0.7-2.7)	0.251	-	-	1.1(0.8-1.6)	0.316	-	-
**Sex**								
(male vs. female)	1.2(0.6-2.4)	0.501	-	-	1.1(0.8-1.7)	0.400	-	-
**Clinical stage**								
(III/IV vs. I/II)	3.7(1.6-8.5)	**0.002**	3.4(1.2-10.4)	**0.031**	1.4(1.0-2.1)	**0.027**	1.4(0.9-2.5)	0.۰95
**Fuhrman grade**								
(III/IV vs. I/II)	4.9(2.3-10.0)	**<0.001**	3.6(1.5-8.6)	**0.003**	1.5(0.9-2.3)	0.05	1.17(0.7-1.8)	0.484
**Tumor size**	2.1(1.2-2.4)	**<0.001**	1.1(1.0-1.2)	**0.018**	1.09(0.9-1.3)	0.355	-	-
**Renal vein invasion**								
(Present vs. Absent)	0.21(0.0-0.4)	<0.001	0.6(0.2-1.6)	0.375	0.8(0.3-2.3)	0.755	-	-
**Microvascular invasion**								
(Present vs. Absent)	0.4(0.2-0.7)	0.008	0.9(0.4-2.2)	0.965	0.4(0.2-0.7)	0.008	0.7(0.4-1.2)	0.347
**Cytoplasmic Twist1 Expression***		0.۱79		0.497		0.105		0.658
Moderate vs. low	0.4(0.2-1.1)	0.084	1.1(0.4-3.0)	0.741	0.5 (0.3-0.9)	0.035	0.7(0.4-1.3)	0.360
High vs. low	0.1(0.2-1.1)	0.121	0.6(0.2-1.7)	0.445	0.7(0.4-1.1)	0.195	0.8(0.4-1.4)	0.494
**Nuclear Twist1 Expression***		0.878				0.900		0.396
Moderate vs. low	0.4(0.2-1.1)	0.142	0.4(0.2-0.8)	0.806	1.3(0.1-9.5)	0.035	0.6(0.3-1.2)	0.184
High vs. low	0.1(0.2-1.1)	0.067	0.4(0.1-1.0)	0.128	1.4(0.1-10.4)	0.195	0.7(0.4-1.4)	0.455

Abbreviations: HR hazard ratio; CI confidence interval
Values in bold are statistically significant.
The variables with P value less than 0.2 were included in multivariable analyses.
*Low expression level is considered as reference group

Association of Twist1 expression with survival outcomes in each subtype of RCC

The main characteristics of patients enrolled for survival analysis according to RCC subtypes is illustrated in table 4. PFS analysis was performed only for ccRCC patients, due to limited number of occurred events in pRCC and ChRCC subtypes.

Table 4. The main characteristics of patients enrolled for survival analysis according to RCC subtypes

**Feature**	**RCC subtype**		
	**ccRCCA**	**ChRCC**	**pRCC**
**Number of patients (N %)**	173(68.)	34(13.5)	45(17.9)
**Mean duration of follow up time(months)(SD)**	47.4(28.4)	40.3 (13.1)	40.3(13.1)
**Disease related death (N %)**	29(74.3)	3(7.6)	7(17.9)
**Recurrence history during follow up (N %)**	3(50.0)	1(16.6)	2(33.3)
**Metastasis history during follow up (N %)**	35(77.7)	3(6.6)	7(15.5)

Alive patients without any complication (N %)	83(69.1)	11(0.9)	26(21.6)
Abbeviations: ccRCC indicates clear cell Renal Cell Carcinoma; ChRCC, chromophob Renal Cell Carcinoma and pRCC, papillary Renal Cell Carcinoma.			

Survival analysis in ccRCC patients

The 5-year PFS for patients whose specimens expressed high cytoplasmic and nuclear levels of Twist1 was 25 and 43%, respectively. In Kaplan–Meier survival analysis, ccRCC patients whose tumors expressed higher cytoplasmic level of Twist1 showed significantly poorer PFS than those with a moderate and low cytoplasmic Twist1 expression (*P* = 0.027). Due to the low number of remaining cases in the group with high nuclear expression of Twist1, survival cures were not exploited. The PFS survival curve according to cytoplasmic Twist1 expression are depicted in Fig.4

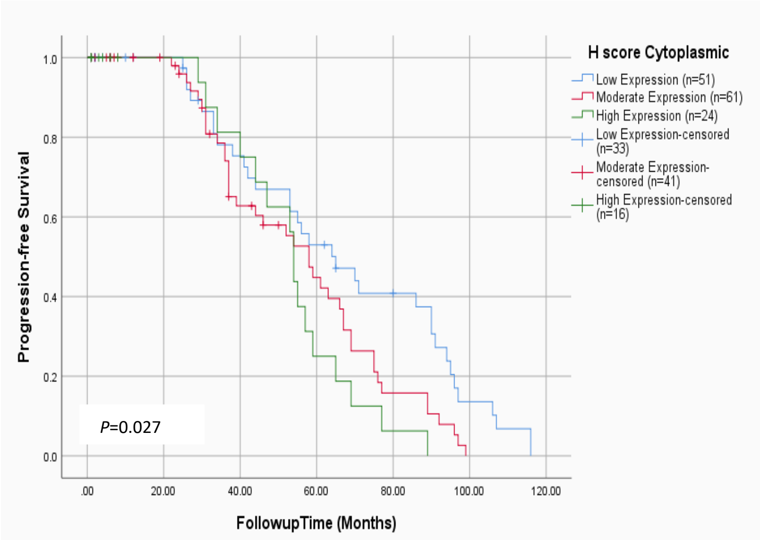

Number at risk by time

		1	2	3	4	5	6	7	8	9
	**Year**	1	2	3	4	5	6	7	8	9
	**Low Cytoplasmic Twist1 expression (N=51)**									
	**N of cumulative Events(censored)**	0	9	19	30	33	-	-	-	-
	**N.at risk**	39	27	15	3	0	-	-	-	-
**ccRCC patients**	**Moderate Cytoplasmic Twist1 expression (N=61)**									
	**N of cumulative Events(censored)**	0	9	18	28	40	41	-	-	-
	**N.at risk**	49	37	25	13	1	0	-	-	-
	**High Cytoplasmic Twist1 expression (N=24)**									
	**N of cumulative Events(censored)**	4	16	-	-	-	-	-	-	-
	**N.at risk**	12	0	-	-	-	-	-	-	-

Figure 4: Correlation between cytoplasmic Twist1 expression and survival rates in patients with clear cell Renal Cell Carcinoma (ccRCC). Progression-free survival (PFS) with cytoplasmic Twist1 expression grouped into low- versus moderate- versus high expression levels.

Univariate and multivariable analyses were performed to assess the clinical significance of various parameters that might influence DSS and PFS in patients with ccRCC.

Clinical stage (HR 3.9, 95% CI 1.6-9.8, *P*= 0.003), Fuhrman nuclear grade (HR 5.4, 95% CI 2.4-12.1, *P*<0.001), and tumor size (HR 1.2, 95% CI 1.1-1.3, *P*<0.001), were significant risk factors affecting the DSS of patients with ccRCC in Univariate analysis. Grade (HR 4.3, 95% CI 1.7-10.6, *P*=0.016), and tumor size (HR 1.13, 95% CI 1.0-1.2, *P*=0.002) were also significant risk factors affecting the DSS of patients with ccRCC in multi variable analysis. There was no significant risk factor in univariate and multivariable analyses using the same variables affecting the PFS of patients with ccRCC.
